# Multi-objective optimisation of polymerase chain reaction continuous flow systems

**DOI:** 10.1007/s10544-022-00610-6

**Published:** 2022-03-22

**Authors:** Foteini Zagklavara, Peter K. Jimack, Nikil Kapur, Osvaldo M. Querin, Harvey M. Thompson

**Affiliations:** 1 School of Computing, University of Leeds, Leeds, LS2 9JT United Kingdom; 2 School of Mechanical Engineering, University of Leeds, Leeds, LS2 9JT United Kingdom

**Keywords:** PCR, Multi-objective Optimisation, Design of Experiments, COMSOL^®^, Microchannel, Pareto Front

## Abstract

A surrogate-enabled multi-objective optimisation methodology for a continuous flow Polymerase Chain Reaction (CFPCR) systems is presented, which enables the effect of the applied PCR protocol and the channel width in the extension zone on four practical objectives of interest, to be explored. High fidelity, conjugate heat transfer (CHT) simulations are combined with Machine Learning to create accurate surrogate models of DNA amplification efficiency, total residence time, total substrate volume and pressure drop throughout the design space for a practical CFPCR device with sigmoid-shape microfluidic channels. A series of single objective optimisations are carried out which demonstrate that DNA concentration, pressure drop, total residence time and total substrate volume within a single unitcell can be improved by up to $\sim$5.7%, $\sim$80.5%, $\sim$17.8% and $\sim$43.2% respectively, for the practical cases considered. The methodology is then extended to a multi-objective problem, where a scientifically-rigorous procedure is needed to allow designers to strike appropriate compromises between the competing objectives. A series of multi-objective optimisation results are presented in the form of a Pareto surface, which show for example how manufacturing and operating cost reductions from device miniaturisation and reduced power consumption can be achieved with minimal impact on DNA amplification efficiency. DNA amplification has been found to be strongly related to the residence time in the extension zone, but not related to the residence times in denaturation and annealing zones.

## Introduction

The Polymerase Chain Reaction (PCR) has revolutionised biological research and diagnostics since its discovery by Kary Mullis in 1983 (Mullis 1990). PCR systems perform a thermal cycling procedure to amplify DNA segments, allowing detection and identification of gene sequences using appropriate optical techniques (Does 2013). They are now used in numerous diagnostic systems, with applications ranging from the rapid detection of infectious diseases (Park et al. 2011) to identification of bacteria causing micro-biologically induced corrosion in oil and gas production systems (Zhu et al. 2006; Agrawal and Lal 2009). An example of the former is the vital role PCR systems are playing in the public health response to COVID-19 (Abbasi-Oshaghi et al. 2020). The PCR thermal cycling procedure consists of the three distinct stages of denaturation, annealing and extension. Denaturation takes place at $\sim$ 95 $^o$C, where the double-stranded DNA denatures into pairs of single-stranded ones. The sample then enters the annealing stage at $\sim$ 56 $^o$C, where the primers form primer-template complexes. The final stage, extension, generally takes place at $\sim$ 70 $^o$C and is where the polymerase binds to the primer-template complexes, catalysing the synthesis of new strands of DNA (Park and Park 2017; Schochetman et al. 1988).

Small, discrete droplets have been used in conventional PCR devices (DBPCR) as separate chemical reactors. The droplets can provide a highly controlled and contaminant-free reaction environment with much smaller thermal mass than in CFPCR systems (Zhang and Jiang 2016). Detailed descriptions of DBPCR systems are given in Ma et al. (2019) and Shi et al. (2020). Despite their advantages, the comparative expense and complexity of DBPCR devices (Zhang and Jiang 2016) have motivated further development and optimisation of single-phase continuous flow PCR (CFPCR) systems, as evidenced by several studies appearing recently (Kaprou et al. 2019; Kulkarni, Goyal et al. 2021; Kulkarni, Salve et al. 2021; Hamad et al. 2021).

Experimental and numerical studies of CFPCR systems have explored how operating and geometry variables affect the thermal cycling process and, ultimately, the efficiency of the DNA amplification process controlling the PCR yield. These have shown that the most influential parameters include the substrate’s thermal conductivity, fluidic channel sizes and spacing, flow rate, while the heating arrangement has also been shown to be very important (Thomas et al. 2014; Chen et al. 2013). Controlling the residence times in each of the thermal zones is also of key importance since insufficient dwell times can reduce PCR yield significantly; Cao et al. (2011) studied the effect of these factors on DNA amplification efficiency DNAAE), both experimentally and numerically. Combining mathematical models of the kinetics of denaturation, annealing and extension processes with models of the flow and thermal processes has proven to be highly beneficial for understanding and hence improving DNAAEs in CFPCR systems (Wang and Li 2010; Cao et al. 2011; Papadopoulos et al. 2015; Zagklavara et al. 2021).

The effect of fluidic channel geometry on PCR performance has been studied widely, with the performance of radial (Schaerli et al. 2009) and spiral (Hashimoto et al. 2004) geometries having been benchmarked against straight channels (Chiou et al. 2001; Frey et al. 2007). The benefits of achieving more uniform flow and thermal conditions have also been explored. The latter was considered experimentally and numerically by Duryodhan et al. (2016), who showed that employing diverging fluidic channels can create more uniform wall temperatures, while Gui and Ren (2006) showed that flow uniformity can be increased through the use of electro-kinetic flow. A number of studies have focused on the influence of heater arrangement, showing that it is important to control the interference and transition times between the thermal zones, and the thermal ‘cross-talk’ between adjacent zones, which can require larger gaps between channels and therefore hinder the drive towards device miniaturisation needed to create portable devices for diagnostic testing purposes (Kumar et al. (2013); Moschou et al. (2014); Papadopoulos et al. (2015); Perwez et al. (2019). Perwez et al. (2019) have recently explored these issues in the context of using a simple, single heater CFPCR chip design. In a similar vein, the lower thermal conductivity of 3D-printable materials has been identified as a major factor limiting its application to CFPCR devices (Park and Park 2017). Furthermore, when it comes to lab-on-chip devices, the pressure drop requirements can become very important, since they often require sophisticated and expensive microfluidic pumps (Fajrial et al. 2021; Ahn et al. 2004) that can be hard to integrate and fabricate (Ahn et al. 2004).

This paper is motivated by the need to develop an effective multi-objective methodology for CFPCR devices. For example, the development of low-cost and rapid diagnostic devices for use in inaccessible regions requires effective device minaturisation and reduced power consumption, whilst maintaining the required rate of DNA amplification. The aim is to provide a powerful means of striking the appropriate balances between the conflicting performance objectives. A simulation-based optimisation methodology is developed, which uses outputs from Computational Fluid Dynamics (CFD) analyses. This approach is now commonplace in many industries, such as the aerospace and automotive ones, with the continued progress in computing power, numerical schemes and design space exploration methods, making it an increasingly powerful means of optimising complex flow systems (Khatir and Thompson 2019). The recent review by Haftka et al. (2016) noted that the number of design variables is key. For large problems with $\mathcal {O}(1000)$ design variables, employing advanced adjoint methods is vital, whereas for CFPCR systems with < 100 design variables, gradient-free surrogate-assisted methods are effective. Important examples of the latter include Gaussian Process Emulators (Domingo et al. 2020), and Moving Least Squares, which is effective at minimising the effects of numerical noise (González Niño et al. 2019). Surrogate modelling using Machine Learning can also be effective for achieving temperature control in CFPCR systems (Lee et al. 2007; Hamad et al. 2021).

The present study applies optimisation methods on a practical CFPCR flow problem, considered recently by Papadopoulos et al. (2015) and Zagklavara et al. (2021). The effect of the PCR protocol on the performance of a CFPCR is investigated in detail, examining the importance of the residence time in each temperature zone (denaturation, extension, annealing). Furthermore, the design approach of doubling the channel width in the extension zone to increase the residence time there (see e.g. Papadopoulos et al. (2015) and Zagklavara et al. (2021)) is also examined, by including it as a design variable. Four objectives of practical interest are studied: the DNA amplification efficiency (DNAAE), the total residence time, the substrate volume and the pressure drop requirements of the unitcell of a microfluidic device. Furthermore, a Pareto front is generated in order to maximise DNAAE, whilst minimising the total residence time and substrate volume of the microfluidic device (Logist et al. 2007; Hashem et al. 2017). Apart from increasing DNAAE, reducing the total substrate volume and total residence time can lead to significant reductions in cost and processing times of the device. Furthermore, the pressure drop is also minimised in order to facilitate the development of microfluidic pumps for lab-on-chip devices, that are often highly sophisticated and expensive (Fajrial et al. 2021; Ahn et al. 2004).

The paper is organised as follows. Section 2 describes the PCR problem of interest while Sect. 3 outlines the conjugate heat transfer problem and mathematical and numerical methods employed. Section 4 presents the results of the numerical simulations and optimisation studies, with conclusions drawn in Sect. 5.

## Problem specification

**Fig. 1:**
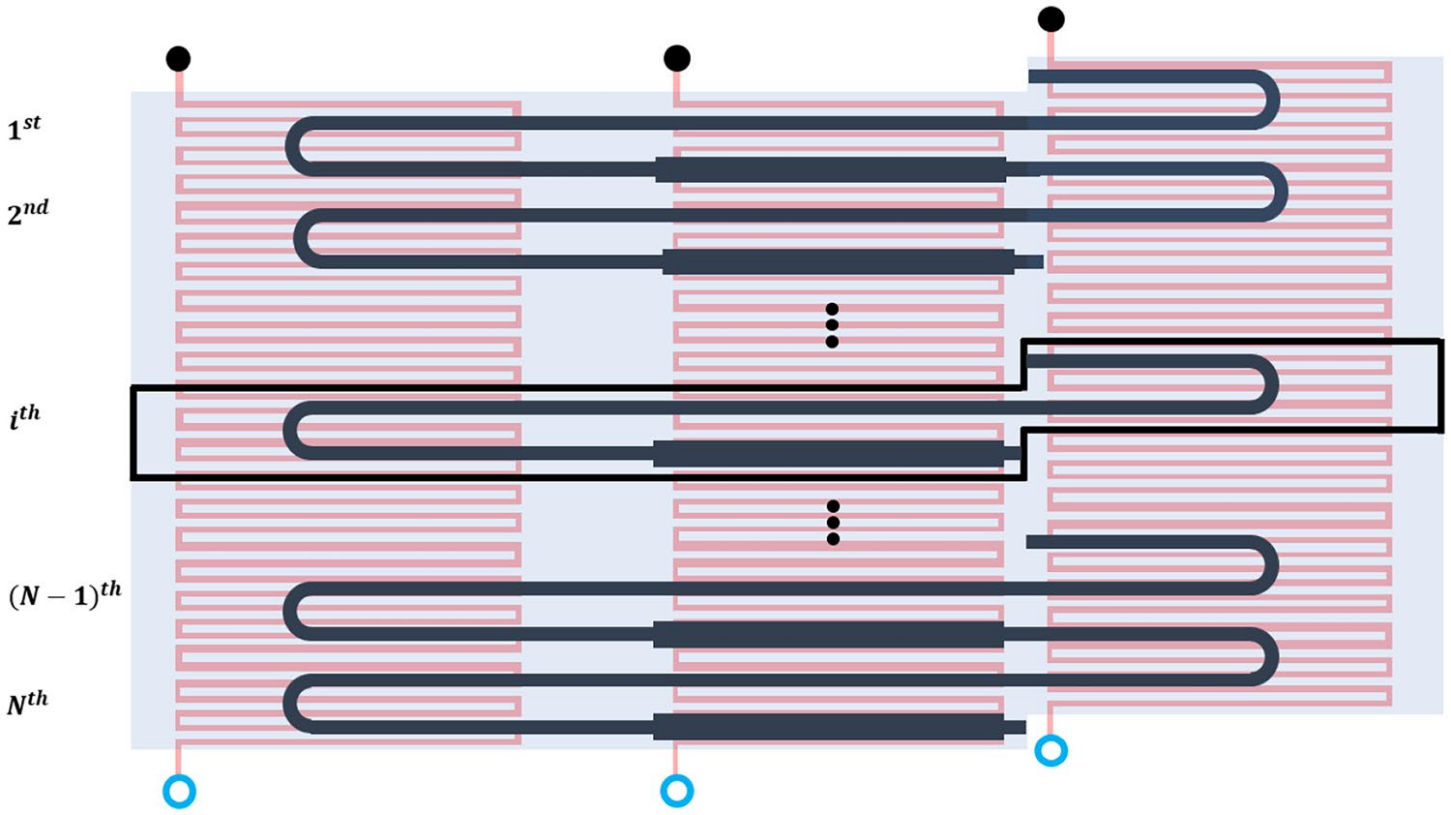
Schematic diagram of the CFPCR device, consisting of *N* cycles or unit-cells (top view)

Within a single PCR cycle, or unit-cell, the temperature of the flowing fluid through the microchannel changes as it passes through three different temperature zones - typically $\sim$95, $\sim$55 and $\sim$72 $^o$C in the denaturation, annealing and extension zones respectively (Papadopoulos et al. 2015). A unit-cell corresponds to one of the *N* PCR cycles that are placed consecutively in a serpentine arrangement, as presented in Fig. 1. The temperature changes along the length of the microchannel are designed to increase the DNA concentration significantly by the time the fluid exits the channel. The cases considered here are based on the chip substrate materials, Kapton, PDMS and PE (Table 1), and the design parameters (Table 2) used in Papadopoulos et al. (2015) and Zagklavara et al. (2021).

**Table 1:** Material properties (Papadopoulos et al. 2015; Moschou et al. 2014)

Material	Heat Capacity	Density	Thermal conductivity	Surface
	[J/(kg K)]	[kg/$m^3$]	[W/(mK)]	emissivity [-]
Copper	$358 + 0.09623384 \cdot T \frac{J}{kg\cdot K}$^a^	8960^a^	401^a^	0.6^b^
Kapton	1090^a^	1420^a^	0.1200^a^	0.78^a^
PDMS	1430^a^	983^a^	0.1511^a^	0.96^a^
PE	2400^a^	950^a^	0.4450^a^	0.92^a^

^a^Papadopoulos et al. (2015)

^b^Moschou et al. (2014)

**Table 2:** Design Parameters of the Microchannel (Papadopoulos et al. 2015)

Parameter^*^	Values	Description
$U_{in}$	from Eq. 4	Average inlet velocity (fully developed)
$Q_{vol}$ $[\mu L /min]$	1.800^*^	Volumetric flowrate
$T_{amb} [K]$	298.15^*^	Ambient temperature
$h [W/(m^2 \cdot K)]$	5^*^	Heat transfer coefficient
$L_{1}$ [*mm*]	4.190^*^	See Fig. 2
$L_{2}$ [*mm*]	0.714^*^	See Fig. 2
$L_{3}$ [*mm*]	0.500^*^	See Fig. 2
$L_{4}$	from Eq. 11	See Fig. 2
$L_{5}$ [*mm*]	1.670^*^	See Fig. 2
$L_{6}$	from Eq. 11	See Fig. 2
$L_{7}$ [*mm*]	1.110^*^	See Fig. 2
$L_{8}$	from Eq. 11	See Fig. 2
$L_{9}$ [*mm*]	0.500^*^	See Fig. 2
$L_{10}$ [*mm*]	3.114^*^	See Fig. 2
$L_{11}$ [*mm*]	2.000^*^	See Fig. 2
$W_{1}$ [*mm*]	2	See Fig. 2
$W_{2}$ $[\mu m]$	400^***^	See Fig. 2
$W_{3}$	from Eq. 12	See Fig. 2
$H_{Kapton}$ $[\mu m]$	100^*^	See Fig. 2
$H_{PDMS}$ $[\mu m]$	50^*^	See Fig. 2
$H_{PE}$ $[\mu m]$	50^*^	See Fig. 2
$H_{Fluid}$ $[\mu m]$	50^***^	See Fig. 2

^*^Values obtained by Papadopoulos et al. (2015), ^*^S.I. units are used for all parameters in all calculations/equations, ^***^Optimum design obtained by Zagklavara et al. (2021)

### Flow modelling

The flow is steady and is governed by the incompressible Navier-Stokes equations (Eqs. 1 and 2).

1 $$\begin{aligned} \rho (\mathbf {u}\cdot \nabla ) \mathbf {u}= \nabla \cdot [ -p \mathbf {I} + \mu ( \nabla \mathbf {u} + (\nabla \mathbf {u})^T)] + \mathbf {F} \end{aligned}$$

2 $$\begin{aligned} \rho \nabla \cdot \mathbf{u} =\mathbf{0} \end{aligned}$$

where $\rho$ is the fluid density, $\mathbf{u}$ the velocity vector, *p*: the pressure, $\mu$ the viscosity and $\mathbf{F}$ the external forces applied to the fluid, such as buoyancy force due to gravitational acceleration, Lorentz forces etc (McDonough 2009; Gerbeau and Le Bris 2000). Flow is laminar since an indicative value of Reynolds number, $Re \sim 0.33$ (Eq. 3) can be calculated for $Q_{vol} = 3 \cdot 10 ^{-11} m^3/s$, $H_{Fluid}$ = 50 $\mu$m, $W_{2}$ = 400 $\mu$m, $t_{R,den}$ = 3s, $t_{R,ext}$ = 6.2s, $t_{R,ann}$ = 4.2s and the fluid properties of water at 72 $^o$C (Rennels and Hudson 2012; Crittenden et al. 2012).

3 $$\begin{aligned} Re = D_h \cdot U_{in} \cdot \rho / \mu = (\frac{2H_{Fluid}W_{2}}{H_{Fluid}+W_{2}})\frac{U_{in}\cdot \rho }{\mu } \end{aligned}$$

where

4 $$\begin{aligned} U_{in} = Q_{vol}/A \end{aligned}$$

5 $$\begin{aligned} A=W_{2} \cdot H_{Fluid} \end{aligned}$$

**Fig. 2:**
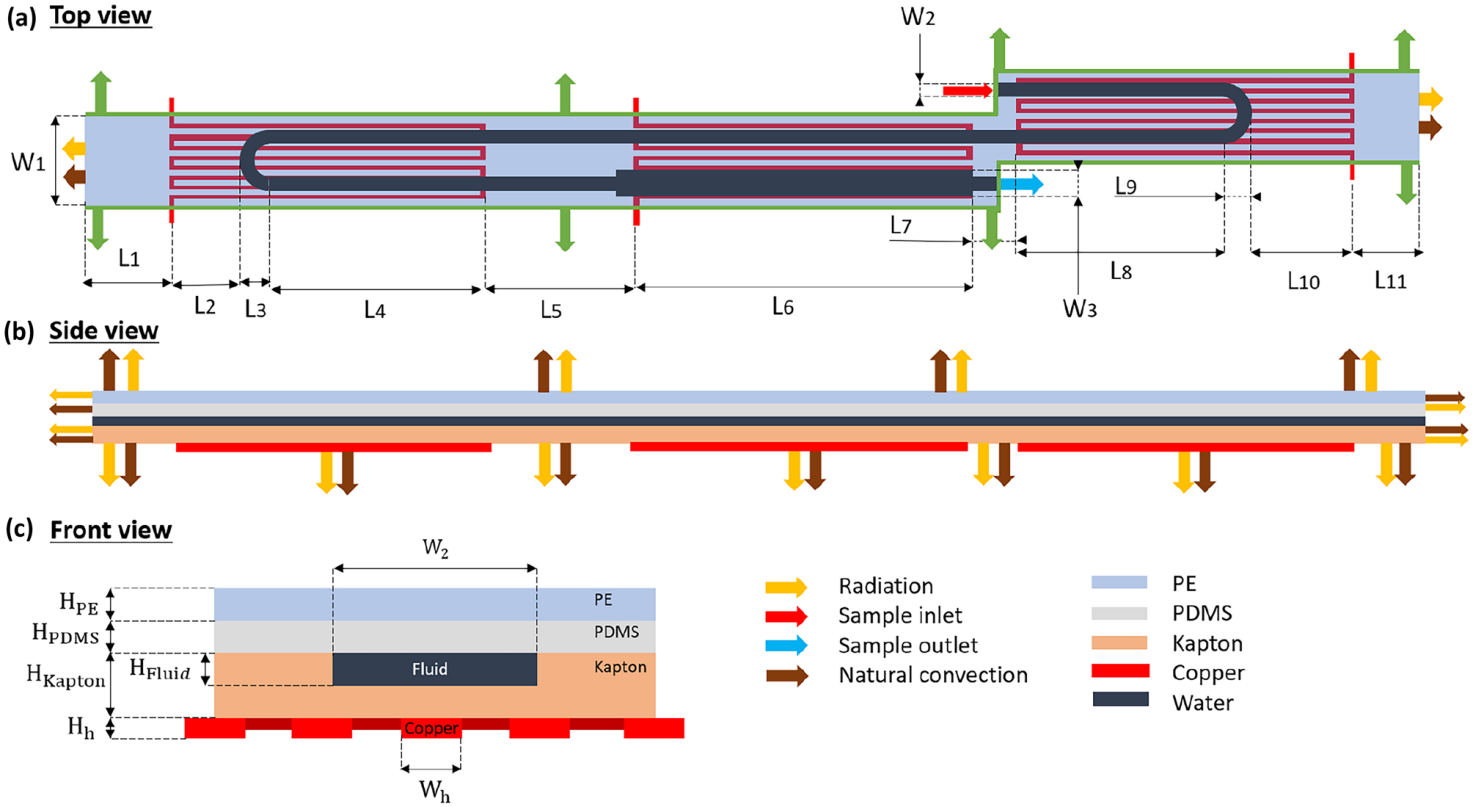
Schematic diagram of the **a**) top, **b**) side and **c**) front view of a unit-cell of CFPCR device, including the boundary conditions applied in each case. The dimensions are presented in Table 2

Equations 1 and 2 are solved on the geometry appearing in Fig. 2, subject to the following boundary conditions: (i) no-slip at the microchannel walls; (ii) fully-developed flow and a value of average inlet velocity, $U_{in}$, at the inlet of the serpentine channel; (iii) zero (relative) pressure at the exit of the microchannel.

### Conjugate heat transfer modelling

Steady state, conjugate heat transfer is modelled via Eq. 6:

6 $$\begin{aligned} \rho C_p (\mathbf {u} \cdot \nabla T) = \nabla \cdot (k \nabla T) +\Sigma Q_{heater,j}+ \Sigma Q_{rad, i} + Q_{nat.conv} \end{aligned}$$

where **u**=**0** everywhere except in the fluid domain. $Q_{heater,j}$ is the heat generation rate of the $j^{th}$ (j = {1, 2, 3}) heater, and is only non-zero at the $j^{th}$ heater-kapton interface. A different heat generation rate is required at each heater to achieve the desired set points of 95, 55 and $72 ^o$C in the denaturation, annealing and extension zones respectively. $Q_{rad,i}$ is the heat flux due to thermal radiation (Eq. 7 (Stefan-Boltzmann law)) of the $i^{th}$ solid substrate (i = {Copper, PDMS, PE, Kapton}), and is only non zero at the outer surfaces of the substrate materials. $Q_{nat.conv}$ is the heat flux due to the heat losses to the ambient, and is given by Eq. 8:

7 $$\begin{aligned} Q_{rad, i}= \epsilon _i \sigma (T_{amb}^4 - T^4) \end{aligned}$$

8 $$\begin{aligned} Q_{nat.conv} = h (T_{amb}-T) \end{aligned}$$

where $T_{amb}$: the ambient temperature, $\epsilon _i$: surface emissivity for solid *i*, $\sigma$: the Stefan - Boltzmann constant and *h*: heat transfer coefficient.

The boundary conditions are applied on the geometry appearing in Fig. 2 as follows: (i) a periodic boundary condition on temperature at the inlet and outlets of the channel; (ii) the heater temperatures at the copper-solid interface in the denaturation, extension and annealing zones are set to $T_{den}$ = 95 $^o$C, $T_{ext}$ =72 $^o$C and $T_{ann}$ = 55 $^o$C, respectively; (iii) periodic temperature boundary conditions at the two sides of the microchannel; (iv) a heat flux of $Q_{nat.conv}$ from Eq. 8 at the top, bottom, front and back sides of the microchannel, due to natural convection; (v) a heat flux of $Q_{rad,i}$ from Eq. 7 at the front, back, top and bottom surfaces of the unit-cell.

### Diluted species modelling

Several kinetic models have been developed for the reactions in PCR systems - see for example those of Hunicke-Smith (1998); Athavale et al. (2001); Aach and Church (2004); Wang and Wang (2010); Papadopoulos et al. (2015) and Chen and Li (2018).

The general equations for the steady state mass conservation of the species are given by Eqs. 9 and 10. The five reactions and the reaction rate constants ($k_A^+, k_A^-, k_D^+, k_D^-, k_E$) considered in this work are presented in Appendix 2, and are described in detail by Papadopoulos et al. (2015).

9 $$\begin{aligned} \nabla \cdot \mathbf {J}_k + \mathbf {u} \cdot \nabla C_k = R_k \end{aligned}$$

10 $$\begin{aligned} \mathbf {J}_k = -{D}_k \nabla C_k \end{aligned}$$

where $C_k$ is the concentration of the $k^{th}$ species (k = {1,2,..,7} corresponding to $S_1S_2$, $S_1$, $S_2$, $P_1$, $P_2$, $S_1P_2$ and $P_1S_2$ respectively (see Appendix 2)), $R_k$ is the reaction rate of the $k^{th}$ species and $D_k$ is the diffusion coefficient of the $k^{th}$ species. The reaction rates are presented in Eqs. 24–30 in Appendix 2, while the diffusion coefficients of the species in the set of Eq. 10 are presented in Table 3 (Papadopoulos et al. 2015).

**Table 3:** Parameter Values (Papadopoulos et al. (2015)

Parameter	Values [$m^2/s$]	Description
$D_{1}$	$10^{-10}$	Diffusion Coefficient of $S_1S_2$ ($c_1$)
$D_{2}$	$10^{-10}$	Diffusion Coefficient of $S_1$ ($c_2$)
$D_{3}$	$10^{-10}$	Diffusion Coefficient of $S_2$ ($c_3$)
$D_{4}$	$10^{-9}$	Diffusion Coefficient of $P_1$ ($c_4$)
$D_{5}$	$10^{-9}$	Diffusion Coefficient of $P_2$ ($c_5$)
$D_{6}$	$10^{-10}$	Diffusion Coefficient of $P_1S_2$ ($c_6$)
$D_{7}$	$10^{-10}$	Diffusion Coefficient of $S_1P_2$ ($c_7$)

The implemented boundary conditions are: (i) no flux at the sides of the microchannel, excluding the inlet and outlet; (ii) initial species concentrations are given in Table 4; (iii) zero inward species flux at the exit of the microchannel ($\mathbf{n} \cdot D_k \nabla C_k$).

**Table 4:** Initial Conditions (Papadopoulos et al. 2015; Wang et al. 2007)

Initial concentration	Values [$mol/m^3$]	Description of the species
$C_{1}$	$5.71\cdot 10^{-9}$	Double - stranded DNA ($S_1S_2$)
$C_{2}$	0	Single - stranded DNA ($S_1$)
$C_{3}$	0	Single - stranded DNA ($S_2$)
$C_{4}$	$3.00 \cdot 10^{-4}$	Single - stranded primer molecule ($P_1$)
$C_{5}$	$3.00 \cdot 10^{-4}$	Single - stranded primer molecule ($P_2$)
$C_{6}$	0	Single-stranded template–primer complex ($P_1S_2$)
$C_{7}$	0	Single-stranded template–primer complex ($S_1P_2$)

## Numerical methodology

The design of the microchannel is based on the design offering the maximum DNA amplification, presented in the publication of Zagklavara et al. (2021). The coupled series of flow, heat transfer and species transport equations described above are solved subject to the boundary conditions using COMSOL Multiphysics 5.4 (COMSOL 2021), as part of the optimisation study. The material properties and the dimensions of the design parameters of the serpentine channel are presented in Tables 1 and 2 respectively. The properties of the fluid are those of water, while the PCR kinetics are described in detail in Appendix 2. The values of the volumetric flowrate at the inlet ($Q_{vol}$), the ambient temperature ($T_{amb}$), the heat transfer coefficient (*h*), the gaps between the three temperature regimes ($L_2$, $L_4$) and the heights of Kapton, PDMS and PE ($H_{Kapton}$, $H_{PDMS}$, $H_{PE}$) are equal to those used by Papadopoulos et al. (2015) (Table 2). Natural heat convection occurs at the walls of the channel, as illustrated in Fig. 2. The ambient temperature and convective heat transfer coefficient are set to $T_{amb}= 25 ^o C$ and $h = 5 W/(m^2 \cdot K)$ respectively. As far as the surface-to-ambient radiation is concerned, the surface emissivity of all materials is presented in Table 1. The channel lengths obtained by Eq. 11 include the 180$^o$ circular arc of $R_{zone} = 500 \mu m$ (when applicable). The effect of the microchannel design variables, $W_2$ and $H_{Fluid}$ on the PCR amplification efficiency and the pressure drop was studied by Zagklavara et al. (2021). According to their results, the [$W_2 (\mu m)$, $H_{Fluid} (\mu m)$] = [400, 50] and [$W_2 (\mu m)$, $H_{Fluid} (\mu m)$] = [400, 80] designs offer the maximum DNA amplification and minimum pressure drop respectively (Zagklavara et al. 2021). As a result, the parameters $W_2$ and $H_{Fluid}$ take the values of 400 $\mu m$ and 50 $\mu m$ respectively, in order to further study designs that offer improved DNAAE.

The values of the residence times ($t_{R,den}$, $t_{R,ext}$ and $t_{R,ann}$) and the three channel lengths in the denaturation, extension and annealing zones vary in each simulation, in order to observe the effect that the PCR protocol has on the objectives of interest. More specifically, $L_4$, $L_8$, and $L_6$ are calculated by Eq. 11 for the different values of $t_{R,den}$, $t_{R,ext}$ and $t_{R,ann}$. Furthermore, $W_3$ is selected as the fourth variable and is defined according to Eq. 12, where $z_{w3}$ is a parameter $\in [0, 1]$. The selection of the fourth variable, $W_3$, is made in order to study the benefit of doubling the width of the microfluidic channel in the extension zone (as originally used by Papadopoulos et al. (2015)).

11 $$\begin{aligned} L_{zone}={\left\{ \begin{array}{ll} \frac{(u_{zone} \cdot t_{R, zone}-\pi R_{zone})}{2} = \frac{Q_{vol} \cdot t_{R, zone} /(W_2 \cdot H_{Fluid})-\pi R_{zone}}{2}, &{} \text {zone= DEN, ANN}\\ u_{zone} \cdot t_{R, zone} = Q_{vol} \cdot t_{R, zone} /(W_3 \cdot H_{Fluid}),&{} \text {zone= EXT} \end{array}\right. } \end{aligned}$$

12 $$\begin{aligned} W_{3}= (z_{w3}+1)W_{2},\quad z_{w3} \in [0, 1], \end{aligned}$$

### Comparisons with Papadopoulos et al. (2015)

The effect of mesh density is considered for the case with $W_2=200 \mu m$ and $H_{Fluid}=50 \mu m$, with five different mesh densities with 163,517, 321,151, 865,781, 4,035,872 and 6,133,359 elements. The Joule Heating model is implemented to describe the function of the copper wire heaters (Appendix 3), as performed by Papadopoulos et al. (2015).

The effect of mesh density on DNA amplification ($log_2$ of the ratio of the average concentration of double stranded DNA at the end of the first cycle to the initial one), pressure drop ($\Delta P$(Pa)) and power consumption of the heaters ($P_h$(W)) is given in Table 5. Table 6 presents the values of the residual errors for the temperature (T), [DNA] and velocity (U) together with the computation times for the five meshes. This shows that the solutions on the mesh with 321,151 elements are effectively mesh independent and all results presented below are obtained on this mesh.·

**Table 5:** Comparison of the $log_2 \frac{[DNA]}{[DNA]_{o}}$, $P_h$ and $\Delta$P for different number of mesh elements

No	Mesh	$log_2 \frac{[DNA]}{[DNA]_{o}}$	deviation	$\Delta$P	deviation	$P_h$	deviation
	Elements	(-)	(%)	(Pa)	(%)	(W)	(%)
1	163,517	0.67	0	288.59	0.91	0.071	0
2	321,151	0.67	0	284.29	−0.59	0.071	0
3	865,781	0.67	0	283.05	−1.02	0.071	0
4	4,035,872	0.67	0	286.01	0.01	0.071	0
5	6,133,359	0.67	0	285.98^*^	0	0.071	0
Papadopoulos et al. (2015)		0.67^*^		Not stated		0.071^*^	

^*^Reference values for calculating the deviation (%)

**Table 6:** Residual Errors and computation times of the three main variables of the system

No	Mesh Elements	Computing time (s)	Residual Error		
			T	[DNA]	U
1	163,517	200	2.545 ⋅ 10^−12^	4.174 ⋅ 10^−37^	2.9513 ⋅ 10^−15^
2	321,151	585	9.6719 ⋅ 10^−13^	1.8469 ⋅ 10^−37^	1.1491 ⋅ 10^−15^
3	865,781	1,557	9.4439 ⋅ 10^−13^	1.4754 ⋅ 10^−37^	9.3432 ⋅ 10^−16^
4	4,035,872	15,096	2.3523 ⋅ 10^−13^	−1.0762 ⋅ 10^−34^	3.3608 ⋅ 10^−16^
5	6,133,359	16,915	1.9196 ⋅ 10^−13^	**−**1.1391 ⋅ 10^−34^	2.8907 ⋅ 10^−16^

**Fig. 3:**
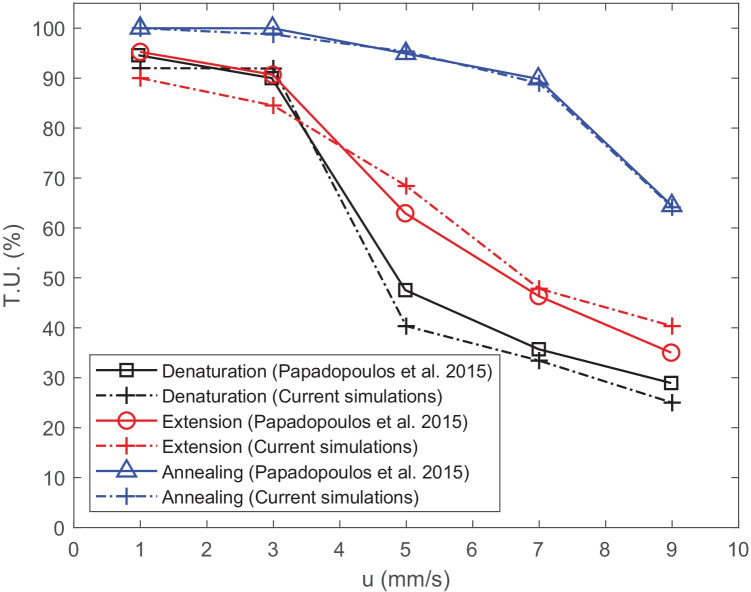
Comparison of the temperature uniformity (% of the zone with fluid temperature within a range of ±1.5 $^o$C from the set point) of the three zones versus inlet velocity with the work of (Papadopoulos et al. 2015)

Comparisons with the results obtained here compared to those of Papadopoulos et al. (2015) show that: (i) $log_2$ of the ratio of the average concentration of double stranded DNA at the end of the first cycle to the initial concentration predicted is the same value, namely 0.67; (ii) the power requirements of the unit cell for performing 1 PCR cycle is also identical, 0.071 W. Note that the indicative power consumption for the denaturation copper wire heater with a rectangular cross-section is calculated as presented in Eqs. 31–36 in Appendix 3. The final comparison is with the temperature uniformity (T.U.) in each temperature regime while varying the inlet velocity, is shown in Fig. 3. The agreement is once again generally very good with the results presented in Papadopoulos et al. (2015). The temperature uniformity values are calculated via Eq. 13.

13 $$\begin{aligned} T.U.(\%)= \frac{\iiint (|T-T_{stp}|<1.5) }{\iiint (T>273.15)},\quad T_{stp} [K]=\{T_{den},T_{ext},T_{ann}\}, \end{aligned}$$

### Optimisation methodology

This optimisation problem focuses on further improving the optimum design of [$W_2$ ($\mu m$), $H_{Fluid}$ ($\mu m$)]$_{log_2(\frac{[DNA]}{[DNA]_o})}$ = [400, 50] presented by Zagklavara et al. (2021), by studying the effect that the implemented PCR protocol (residence times) and one additional geometrical parameter ($W_3$) can have on performance objectives of interest in a unitcell. Each unitcell is identical to the next, apart from the species concentrations - hence the periodic boundary conditions mentioned in Sect. 2.2. As a result, improving the performance of 1 unitcell leads to the improvement of all the cycles and hence the entire device.

More specifically, the effect of residence times in the denaturation ($t_{R,den}$), extension ($t_{R,ext}$) and annealing ($t_{R,ann}$) zones together with the channel width in the extension zone ($W_3$) is investigated on the DNA amplification, pressure drop, total residence time and total substrate volume. The channel lengths are adjusted via Equation 11 to achieve the values of $t_{R,den}$, $t_{R,ext}$ and $t_{R,ann}$. A surrogate-enabled approach is adopted and design of experiments is performed. The ranges of the residence times are created by $t_{R,zone}|_{(Papadopouloset al., 2015)} \pm 1.5s$ (Table 7). The range of the fourth design variable, $W_3$, is set at 400–800 $\mu m$ in order to examine the benefits of increasing (up to twice the width of the microchannel in the other zones, $W_2$) the width in the extension zone in particular, as performed by Papadopoulos et al. (2015). The material properties and the dimensions of the design parameters of the channel are presented in Tables 1 and 2 respectively, and are based on the design proposed by Papadopoulos et al. (2015).

**Table 7:** Upper and lower boundaries of the variables used

No	Variables	Unit	Range
1	$t_{R,den}$	*s*	[1.5–4.5]
2	$t_{R,ext}$	*s*	[4.7–7.7]
3	$t_{R,ann}$	*s*	[2.7–5.7]
4	$z_{w3}$ (or $W_3$)	- (or $\mu m$)	[0–1] (or [400–800])

The objective functions considered are obtained from the dimensionless measurement of the DNA amplification ($log_2 (\frac{[DNA]}{[DNA]_o}) (-)$, where [*DNA*] is the average DNA concentration at the end of the channel and $[DNA]_o$ the initial DNA concentration), the unitcell pressure drop along the microchannel ($\Delta$P (*Pa*)), the total unitcell residence time ($t_{R,total}$ (*s*)) and total unitcell substrate volume ($V_{s,total}$ ($m^3$)). More specifically, COMSOL Multiphysics is used to obtain the values of the four objectives, which are then non-dimensionalised ($obj_1$, $obj_2$, $obj_3$ and $obj_4$ for $-log_2 \frac{[DNA]}{[DNA]_o}$, $\Delta$P, $t_{R,total}$ and $V_{s,total}$ respectively) (scaled to lie between 0-1) in the generated metamodels. $obj_1$ is defined as the negative of $-log_2 \frac{[DNA]}{[DNA]_o}$ in order to switch to four minimisation studies. The Morris Mitchel Latin Hypercube method is used to generate 160 sampling points, using code based on the work of Julie (2012), after modifying it to include the sixteen corner points of the design domain. The 160 sampling points are presented in Appendix 1. The computational model is then evaluated at the 160 sampling points and metamodels for the four objective functions are created using Neural Networks (NN).

#### Development of metamodels

As far as the metamodels are concerned, feed-forward NNs (Leijnen and Veen 2020) and Levenberg-Marquardt back-propagation are used for data fitting, based on the matlab function *fitnet* (MathWorks 2020b). The Mean Squared Error (MSE) performing function is selected together with k-fold evaluation (Manriquez-Sandoval 2021), to test and improve the quality of the NNs. The k-fold method is often used for the evaluation of the performance of classification algorithms, especially for larger datasets (Wong 2015). Such an example is the work of Abellán-García (2021), that used the k-fold validation method to train an artificial neural network with one hidden layer.

The effects of the number of hidden layers, together with the % of testing and training data are investigated for each objective function. NNs with small numbers of hidden layers ([2], [4]) are found to be unable to describe the behaviour of the system adequately, leading to the failure of the optimisation algorithms in obtaining the optimal design solutions. The [4,4] setup for the hidden layers is selected, since it offers $<O(10^{-5})$ accuracy in the prediction of the optimum designs. Table 8 presents the designs offering low values of MSE ($<O(10^{-5})$).

**Table 8:** Details of the NNs used for the four objective functions

Objective Function	No of k-folds	Testing data (%)	Training data (%)	No of hidden layers	MSE
1	8	12.5	87.5	[4,4]	$O(10^{-6})$
2	9	11.1	88.9	[4,4]	$O(10^{-7})$
3	9	11.1	88.9	[4,4]	$O(10^{-7})$
4	9	11.1	88.9	[4,4]	$O(10^{-6})$

#### Optimisation

The *e05jbc* function of the NAG optimisation library (NAG 2020), which is based on the Multi-level Coordinate Search method described in Huyer and Neumaier (1999), uses the meta-models of the objectives to solve the optimisation problems. Subsection 4.1 presents the metamodels for $obj_1$, $obj_2$, $obj_3$ and $obj_4$. Subsection 4.2 then describes the optimisation method used to locate the optimum values for the four objectives, which is based on the *e05jbc* NAG routine (NAG (2020)). Furthermore, the results of a multi-objective optimisation study are also presented in the form of a Pareto front, showing the available compromises between competing objectives.

## Results

### Response surfaces

**Fig. 4:**
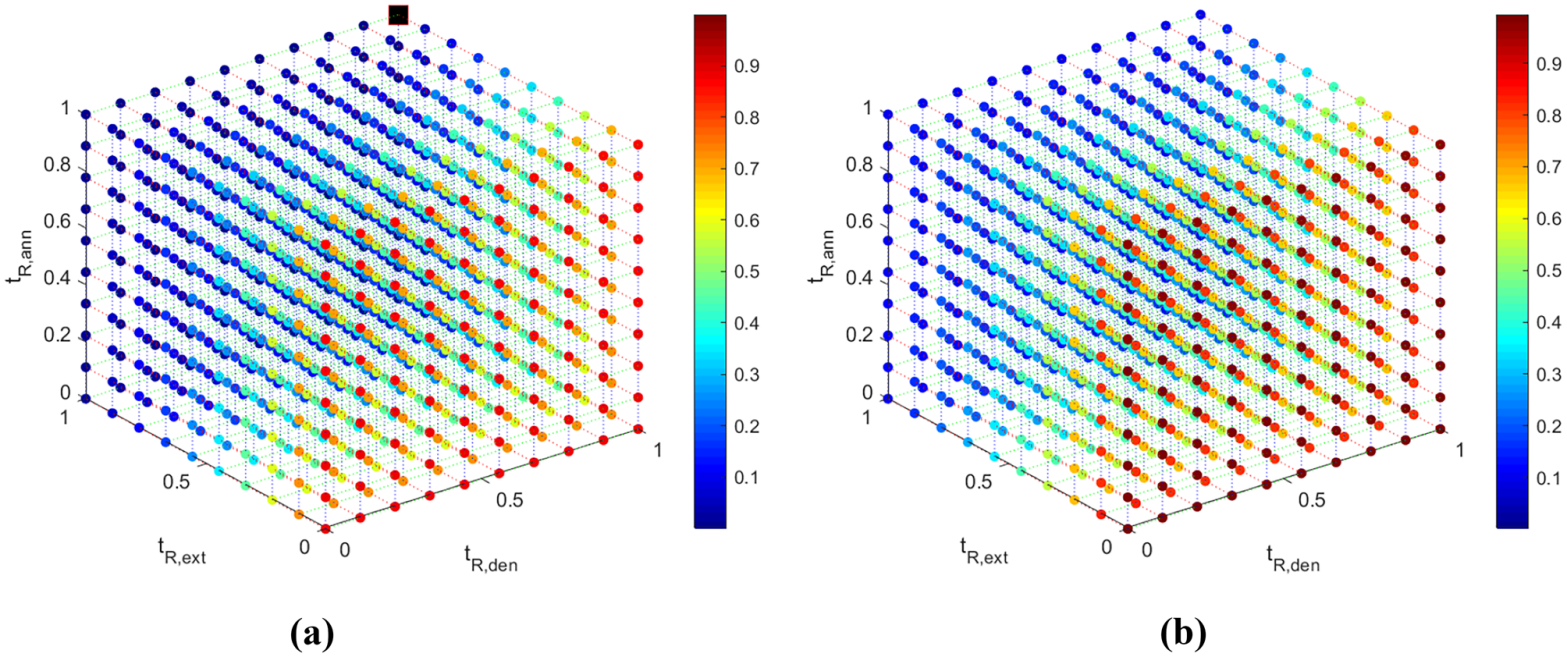
Visual representation of the $log_2(\frac{[DNA]}{[DNA]_o})$(-) data (colorbar) for (**a**) $z_{w3}$ = 0 and (**b**) $z_{w3}$ = 1. The optimum solution is presented in a black square in Fig. 4a

**Fig. 5:**
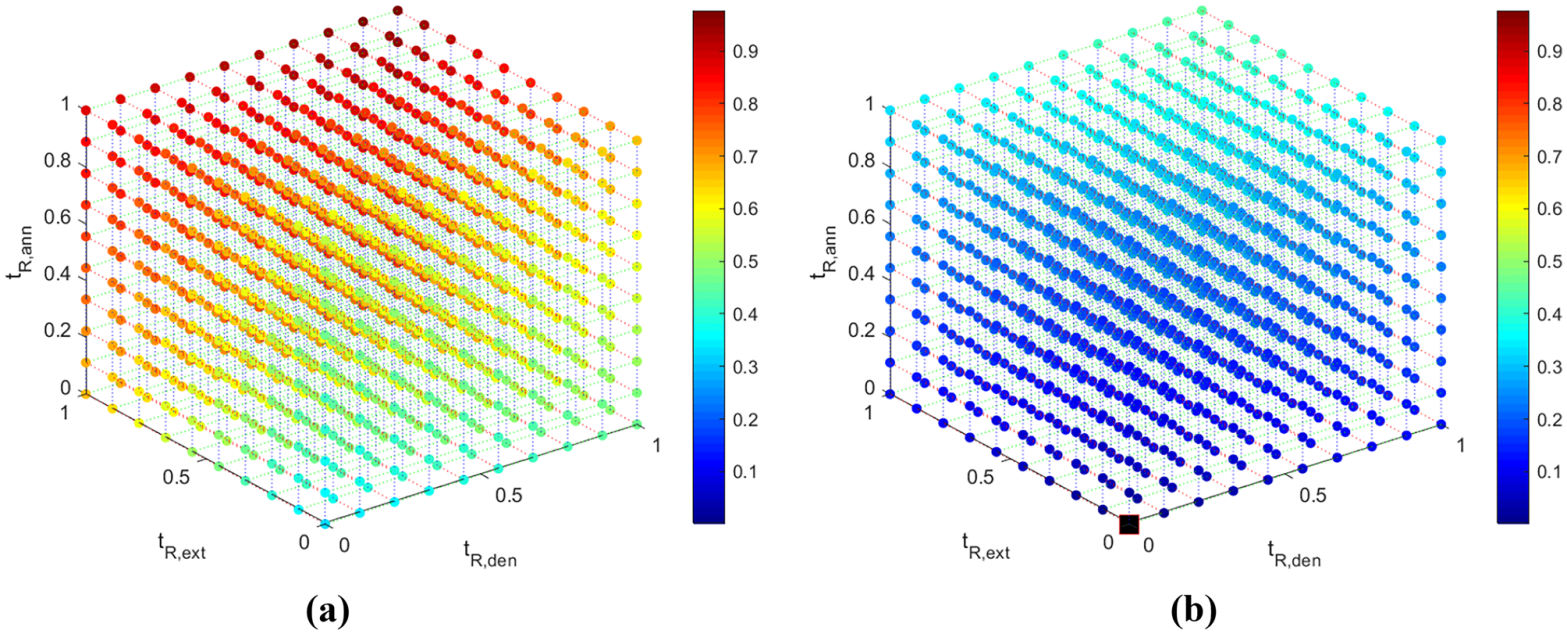
Visual representation of the $\Delta p$(-) data (colorbar) for (**a**) $z_{w3}$ = 0 and (**b**) $z_{w3}$ = 1. The optimum solution is presented in a black square in Fig. 5b

**Fig. 6:**
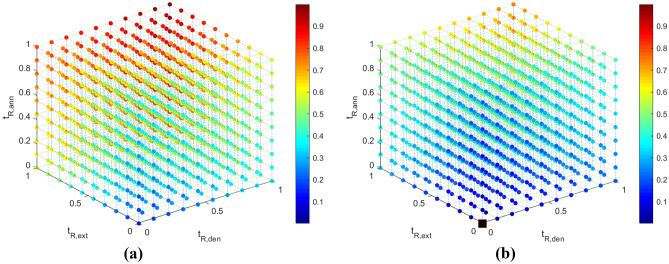
Visual representation of the $t_{R,tot}$(-) data (colorbar) for (**a**) $z_{w3}$ = 0 and (**b**) $z_{w3}$ = 1. The optimum solution is presented in a black square in Fig. 6b

**Fig. 7:**
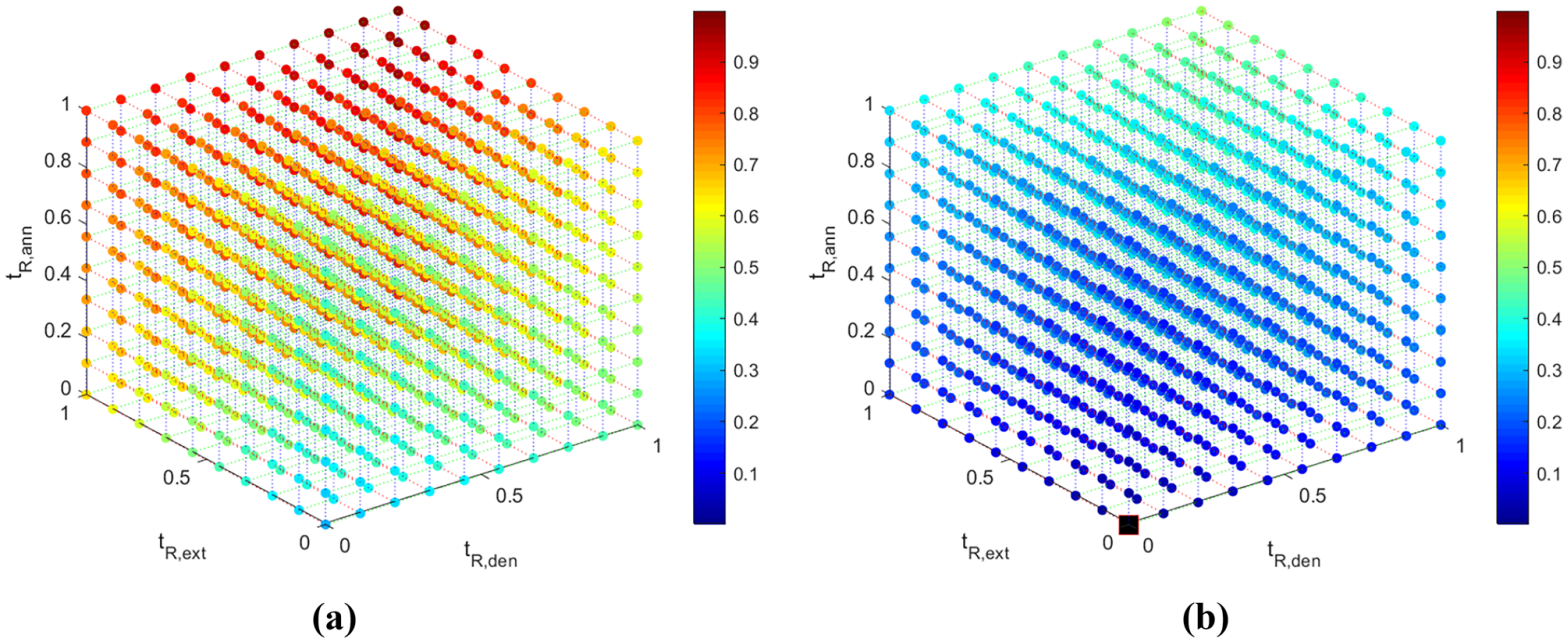
Visual representation of the $V_{S,tot}$(-) data (colorbar) for (**a**) $z_{w3}$ = 0 and (**b**) $z_{w3}$ = 1. The optimum solution is presented in a black square in Fig. 7b

The *fitnet* matlab function is used to generate the NNs for $-log_2(\frac{[DNA]}{[DNA]_o})$, $\Delta p$, $t_{R, tot}$ and $V_{S, tot}$. The values of the objectives are scaled appropriately between 0-1 (see Appendix 6). The 3D response surfaces (for constant values of $z_{w3}$) are developed using the libraries presented by Zhivomirov (2021), and are presented in Figs. 4, 5, 6 and 7 for $log_2(\frac{[DNA]}{[DNA]_o})$, $\Delta p$, $t_{R, tot}$ and $V_{S, tot}$ respectively. The colorbar is used to present the values of the objectives, while a 3D response surface is printed for a different value of the fourth design variable, $z_{w3}$. The sampling data points used to create the response surfaces are provided in Table 14 of Appendix 1. Appendix 5 presents the response surfaces for all four objectives for more values of $z_{w3}$.

The correlation coefficients between the DNA amplification - total residence time and the DNA amplification - individual residence times are given in Table 9. $log_2\frac{[DNA]}{[DNA]_{o}}$ and $t_{R,ext}$ appear to be strongly related, while there appears to be very little correlation between $log_2\frac{[DNA]}{[DNA]_{o}}$ - $t_{R,den}$ and $log_2\frac{[DNA]}{[DNA]_{o}}$ - $t_{R,ann}$. The $log_2\frac{[DNA]}{[DNA]_{o}}$ also appears to not be significantly related to the width of the channel in the extension zone.

**Table 9:** Correlation Coefficients

Function	$t_{R,den}$	$t_{R,ext}$	$t_{R,ann}$	$t_{R,tot}$	$z_{w3}$
$log_2\frac{[DNA]}{[DNA]_{o}}$	−0.08	0.98	−0.01	0.72	−0.16

### Optimisation

#### Single-objective studies

As part of the single-objective studies, the minima of the metamodels of $obj_1$, $obj_2$, $obj_3$ and $obj_4$ are found at [$t_{R,den}$ (*s*), $t_{R,ext}$ (*s*), $t_{R,ann}$ (-), $z_{w3}$ (-)]$_{obj_1}$ = [4.5, 7.7, 5.7, 0], [$t_{R,den}$ (*s*), $t_{R,ext}$ (*s*), $t_{R,ann}$ (-), $z_{w3}$ (-)]$_{obj_2}$ = [1.5, 4.7, 2.7, 1], [$t_{R,den}$ (*s*), $t_{R,ext}$ (*s*), $t_{R,ann}$ (-), $z_{w3}$ (-)]$_{obj_3}$ = [1.5, 4.7, 2.7, 1] and [$t_{R,den}$ (*s*), $t_{R,ext}$ (*s*), $t_{R,ann}$ (-), $z_{w3}$ (-)]$_{obj_4}$ = [1.5, 4.7, 2.7, 1] respectively. These designs are then tested including the Joule Heating model. Details of these designs and the values of the objectives can be found in Tables 10 and 11 respectively. The optimum designs of $obj_1$, $obj_2$, $obj_3$ and $obj_4$ are presented in Figs. 4a, 5b, 6b and 7b respectively.

**Table 10:** Details of the designs appearing in Fig. 8 and Table 11

Design	Reference	Objective to opt	$W_2$	$W_3$	$H_{Fluid}$	$t_{R, den}$	$t_{R, ext}$	$t_{R, ann}$
			($\mu m$)	($\mu m$)	($\mu m$)	(*s*)	(*s*)	(*s*)
1	P	-	200	400	50	3.0	6.2	4.2
2	CW	-	200	400	50	3.0	6.2	4.2
3	Z	$obj_1$	**400**	800	**50**	3.0	6.2	4.2
4	CW	$obj_1$	400	**400**	50	**4.5**	**7.7**	**5.7**
5	Z	$obj_2$	**400**	800	**80**	3.0	6.2	4.2
6	CW	$obj_2$, $obj_3$, $obj_4$	400	**800**	50	**1.5**	**4.7**	**2.7**

The design variables in each study are presented in **bold**

*CW* Current Work, *Z* (Zagklavara et al. (2021)), *P* Papadopoulos et al. (2015)

**Table 11:** Optimum solutions obtained with e05jbc NAG routine for$log_2 \frac{[DNA]}{[DNA]_{o}}$,$\Delta P$,$t_{R, tot}$and$V_{S, tot}$. Details of Designs 1-6 can be found in Table 10

Design	$log_2 (\frac{[DNA]}{[DNA]_o})^*$		$\Delta P^*$		$t_{R, tot}^*$		$V_{S, tot}^*$	
	(−)	($\%$)	(*Pa*)	($\%$)	(*s*)	($\%$)	($m^3$)	($\%$)
1	0.67	-	-	-	-	-	-	-
2	0.67	0.00	284.29	-	18.47	-	1.22 ⋅ 10^−8^	-
3	0.70	4.48	74.88	−73.66	-	-	8.12 ⋅ 10^−9^	−33.45
4	0.78	16.42	140.62	−50.54	29.31	58.71	1.15 ⋅ 10^−8^	−5.62
5	0.62	−7.46	13.74	−95.17	-	-	7.62 ⋅ 10^−9^	−37.51
6	0.58	−13.43	55.44	−80.50	15.18	−17.80	6.92 ⋅ 10^−9^	−43.23

^*^All the values are calculated using the Joule Heating module. The deviations are calculated based on Design 2

**Fig. 8:**
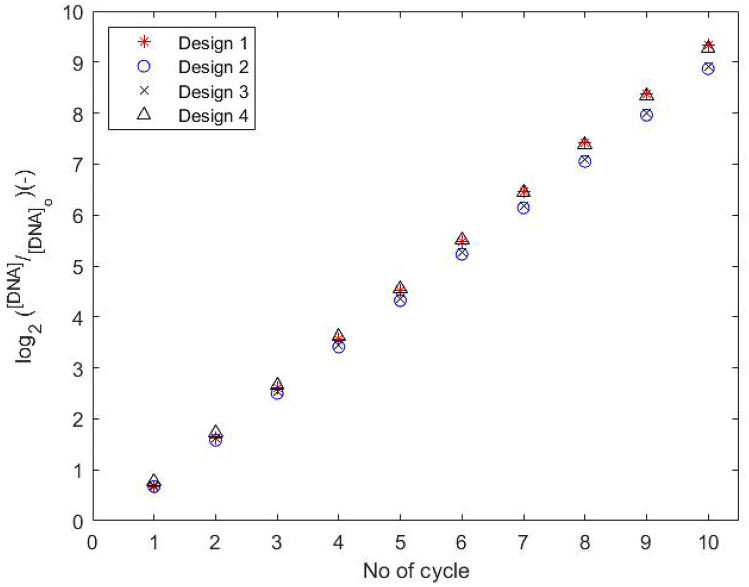
Values of $log_2 (\frac{[DNA]}{[DNA]_o}) (-)$ for 10 PCR cycles. The details of the four designs are presented in Table 10. Designs 1 and 2 present the designs by Papadopoulos et al. (2015) and its validation (current work). Designs 3 (Zagklavara et al. (2021) and 4 (current work) present the designs offering maximum DNA amplification

Design 4 (see Table 10) offers a 16.42% increase in the value of $log_2\frac{[DNA]}{[DNA]_{o}}$ (or $\sim 5.7\%$ increase in [DNA]) and 50.54% and 5.62% decrease in the values of pressure drop and total substrate volume respectively, from the corresponding values obtained for the [$W_2$ ($\mu m$), $H_{Fluid}$ ($\mu m$)]=[200, 50] design of Papadopoulos et al. (2015). In order to examine the significance of the $\sim 5.7\%$ increase in [DNA] for a single unitcell, ten consecutive PCR cycles are simulated for Designs 2 and 4 (see Table 16, Appendix 4), using the Joule Heating model for the function of the copper wire heaters. The results are presented in Fig. 8. According to the data obtained for Design 4, this $\sim 5.7\%$ increase in [DNA] in the first PCR cycle, is expected to increase the concentration of DNA approximately by $\sim 32 \%$ in ten cycles (compared to Design 2). Furthermore, by offering a $\sim 51 \%$ reduction in the pressure drop requirements, the operating cost of such device is expected to be reduced significantly. However, this design also leads to an increase of 58.7% in the total residence time.

**Fig. 9:**
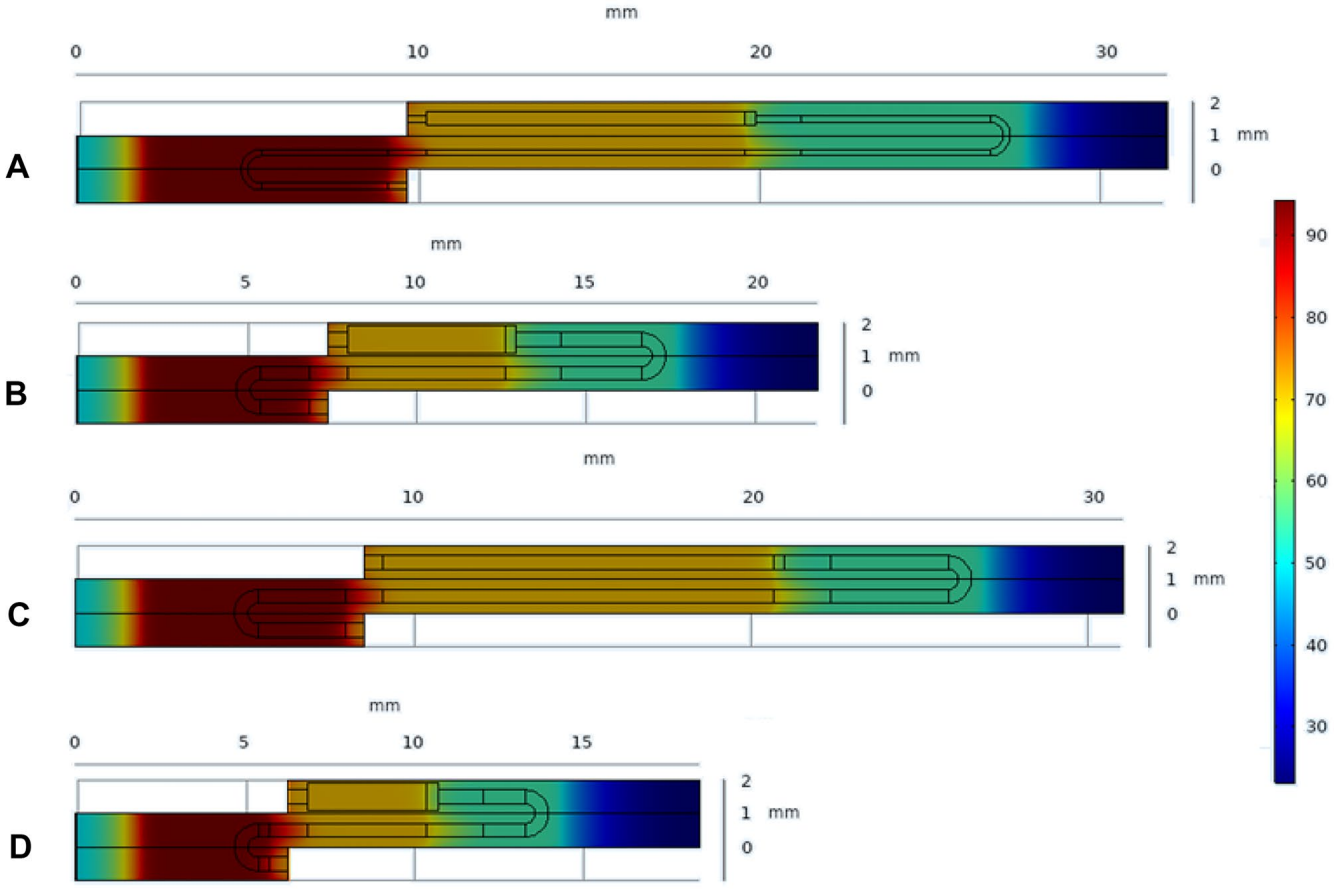
Temperature profiles of the different unitcell designs presented in Tables 10 and 11: **A**) Design 2, **B**) Design 3, **C**) Design 4, **D**) Design 6

**Fig. 10:**
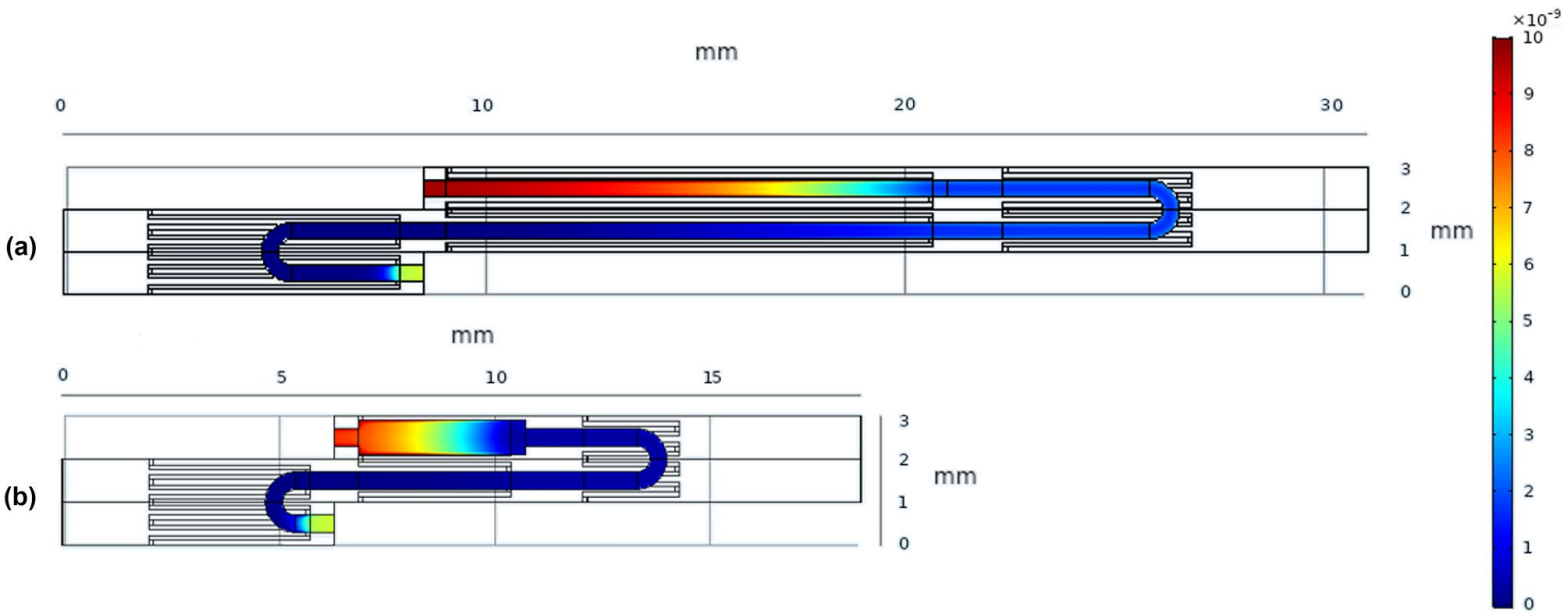
DNA concentration profiles at the centreline along the length of the microchannel of **a**) Design 4 and **b**) Design 6 of the unitcell, presented in Tables 10 and 11

On the other hand, Design 6 (see Table 10) leads to a 80.50%, 17.80% and 43.23% decrease in the values of pressure drop, total residence time and total substrate volume respectively. However, this design also comes with a 13.43 % decrease in $log_2\frac{[DNA]}{[DNA]_{o}}$ (or a 6.6% decrease in the [DNA]) compared to the one presented by Papadopoulos et al. (2015). Figure 9 presents a comparison between the different unitcell designs, together with their temperature profiles. Figure 10 shows the DNA concentration profiles at the middle plane in the fluid domain for the two designs optimising the four objectives.

#### Multi-objective study

**Fig. 11:**
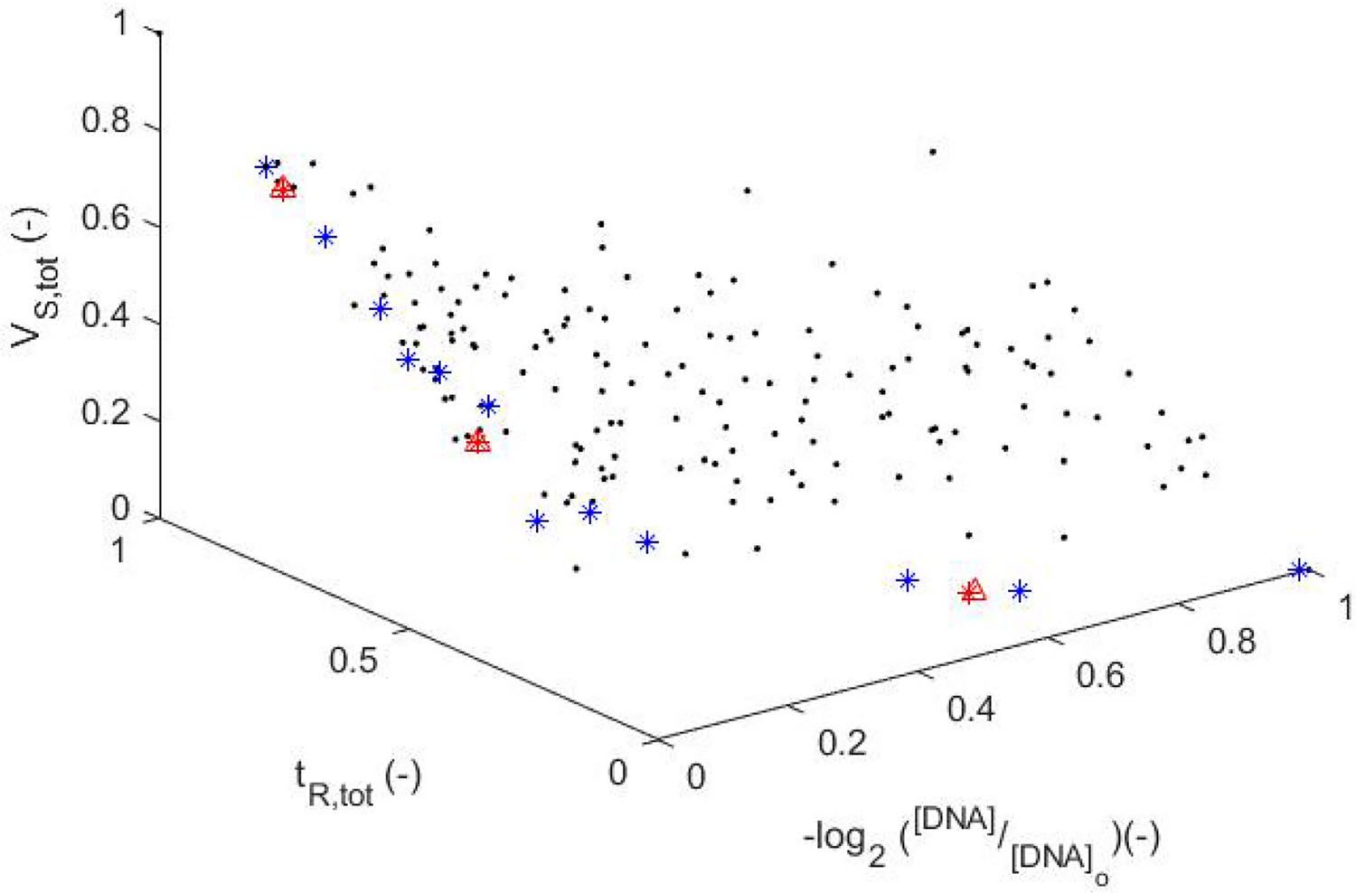
Pareto front (star points) generated with *gamultiobj* (MathWorks 2020) of Matlab, for the $-log_2 (\frac{[DNA]}{[DNA]_o})$ ($obj_1$), the $t_{R,tot}$ ($obj_3$) and the $V_{S,tot}$ ($obj_4$). Three points of the Pareto front (red star points) are validated (red triangle points) (see Tables 12 and 13). The black dots and red triangles represent high fidelity data obtained using COMSOL Multiphysics

The single-objective optimisation results show that conflicts between the objectives results in a complex multi-objective design problem. For the purposes of visualisation, three out of the four objectives ($log_2\frac{[DNA]}{[DNA]_{o}}$, $t_{R,tot}$, $V_{S,tot}$) are selected within a multi-objective optimisation to generate a Pareto front (Fig. 11). The Pareto front is hence a 3D plot, that is developed using the *gamultiobj* function (MathWorks 2020a), in order to demonstrate the available compromises between the three objectives. The values of $obj_1$, $obj_3$ and $obj_4$ are dimensionless and scaled between 0 and 1, to aid visualisation of the multi-objective results. The values of *FunctionTolerance* and *MaxGenerations* are adjusted to $1\cdot 10^{-6}$ and $N_{DVARS} \cdot 200$, where $N_{DVARS}$ is the number of design variables ($N_{DVARS}$ = 4). Three of the optimal solutions in the Pareto front plot are validated using the simulation model (Tables 12 and 13), deviating less than $\sim 0.15 \%$ for all three cases. The Pareto front offers the ability to significantly ameliorate the performance of the device depending on the requirements of the designer/engineer. For example, the design of Point 2 appearing in Tables 12 and 13, illustrates the ability to improve $t_{R,tot}$ and $V_{S,tot}$ by 24.64% and 25.75% respectively when compromising on $log_2\frac{[DNA]}{[DNA]_{o}}$ by only 2.22%.

**Table 12:** Validation of three points appearing in the Pareto-front plot (Fig. 11)

Point	$t_{R, den}$		$t_{R, ext}$		$t_{R, ann}$		$z_{w3}$	$W_{3}$
	(−)	(*s*)	(−)	(*s*)	(−)	(*s*)	(-)	($\mu m$)
1	0.0747	1.72	0.9974	7.69	0.9611	5.58	0.0544	421.76
2	0.0259	1.58	0.9817	7.65	0.621	4.56	0.7257	690.28
3	0.0037	1.51	0.3211	5.66	0.0121	2.74	0.9923	796.92

**Table 13:** Validation of three points appearing in the Pareto-front plot (Fig. 11)

	Pareto Optimum			Model			Deviation		
Point	O1	O3	O4	O1	O3	O4	O1	O3	O4
	(-)	(s)	($m^3$)	(-)	(s)	($m^3$)	(%)	(%)	(%)
1	0.770	26.014	1.050 ⋅ 10^−8^	0.770	26.014	1.050 ⋅ 10^−8^	0.00	0.00	0.01
2	0.755	22.083	8.546 ⋅ 10^−9^	0.755	22.084	8.546 ⋅ 10^−9^	0.00	0.00	0.00
3	0.674	16.680	7.187 ⋅ 10^−9^	0.673	16.681	7.187 ⋅ 10^−9^	0.15	−0.01	0.00

*O1* $$log_2\frac{[DNA]}{[DNA]_{o}}$$, *O3* $$t_{R,tot}$$, *O4* $$V_{S,tot}$$

## Conclusion

The development of practical CFPCR devices offers a complex, multi-objective design challenge due to the conflicts between the required DNA amplification and other practical constraints, such as manufacturing and operating costs related to size and power consumption. The latter are particularly important for low-cost devices targeted at lower-income countries. This paper has developed an effective multi-objective optimisation methodology which allows designers to strike an appropriate balance between the various competing objectives. The methodology uses a series of high fidelity CHT simulations which also account for the kinetics of the DNA amplification to predict the DNA amplification efficiency. As a basis for the chip device, the width and height of the microchannel are constant and (along with parameters of volumetric flowrate, gap lengths and material properties) are consistent with the work of Zagklavara et al. (2021).

Results indicate that doubling the width of the microchannel in the extension zone, together with the residence time in denaturation and annealing zones does have significant effect on the DNA amplification. The residence time in extension zone however has been found to be strongly related to the DNA amplification. From consideration of the Pareto front, several designs are presented, and depending on design priorities, different design solutions can be used to improve the designs of Papadopoulos et al. (2015) and Zagklavara et al. (2021). The Pareto front includes designs ranging from those with low DNA amplification, low total device volume and operation time to high values of the DNA amplification, high total device volume and operation time or design compromises between the three objectives.

The first types of design offers the ability to reduce the total material volume, operation time and pressure drop requirements by up to $\sim 43.2\%$, $\sim 17.8\%$ and $\sim 80.5\%$ respectively. However, such design modifications can lead up to $\sim 6.6\%$ reduction in the [DNA] in the a unitcell. Single objective optimisation on the DNAAE, shows that it is possible to increase DNA concentration by up to $\sim 5.7\%$ in the first PCR cycle, which simulations show results in an increase of $\sim 32\%$ over ten PCR cycles. At the same time, this design offers a reduction in the total pressure drop ($\sim 50.5\%$) together with a small reduction in the material volume ($\sim 5.6\%$), having however a $\sim 58.7\%$ increase in the total operating time. According to the results obtained, all designs have the potential to minimise pumping requirements for such devices; with reductions in pressure drop allowing for smaller pumps to be used (particularly when building integrated lab-on-chip devices). The smaller size and reduced pumping requirements also minimise power requirements, which is an important consideration when these are used within handheld devices containing their own power-sources. This supports the ongoing efforts to develop field-ready microfluidic systems.

Future research directions include comprehensive experimental validation of the optimisation results, and their extension to a wider range of design variables.

## Figures and Tables

**Fig. 12:**
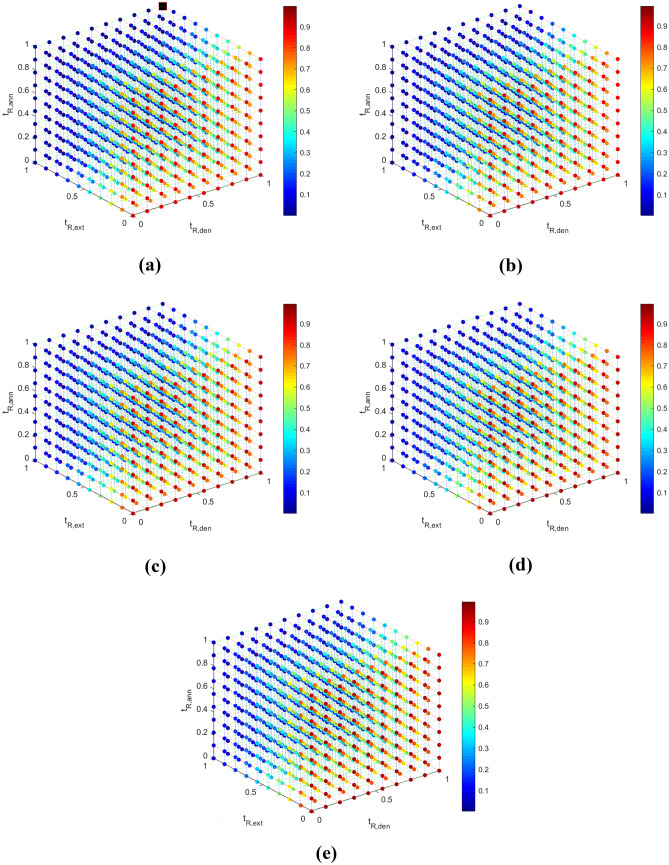
Visual representation of the $log_2 \frac{[DNA]}{[DNA]_{o}}$ (−) data (colorbar) for (**a**) $z_{w3}$ = 0, (**b**) $z_{w3}$ = $0.\bar{1}$, (**c**) $z_{w3}$ = $0.\bar{2}$, (**d**) $z_{w3}$ = $0.\bar{3}$, (**e**) $z_{w3}$ = $0.\bar{4}$

**Fig. 13:**
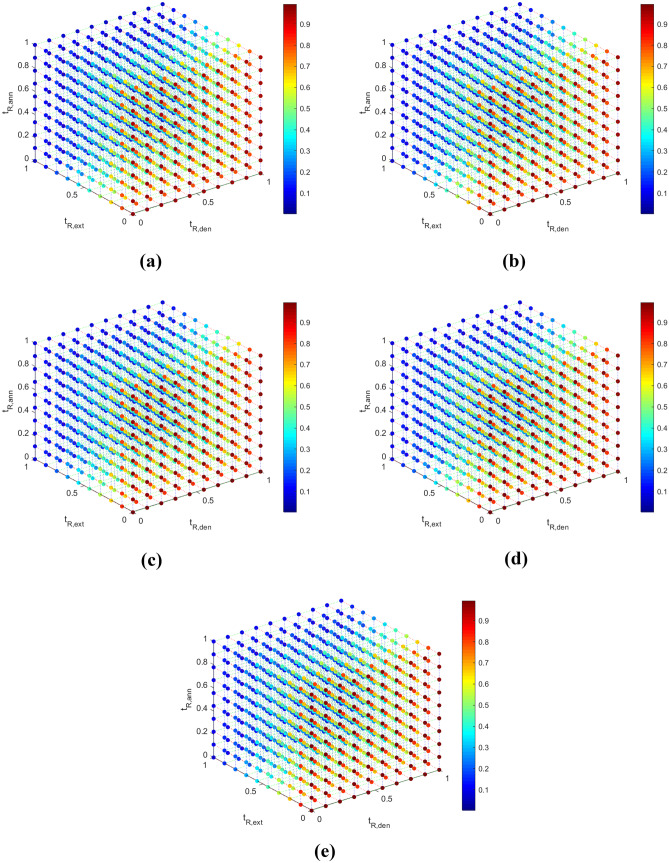
Visual representation of the $log_2 \frac{[DNA]}{[DNA]_{o}}$ (−) data (colorbar) for (**a**) $0.\bar{5}$, (**b**) $z_{w3}$ = $0.\bar{6}$, (**c**) $z_{w3}$ = $0.\bar{7}$, (**d**) $z_{w3}$ = $0.\bar{8}$, (e) $z_{w3}$ = 1

**Fig. 14:**
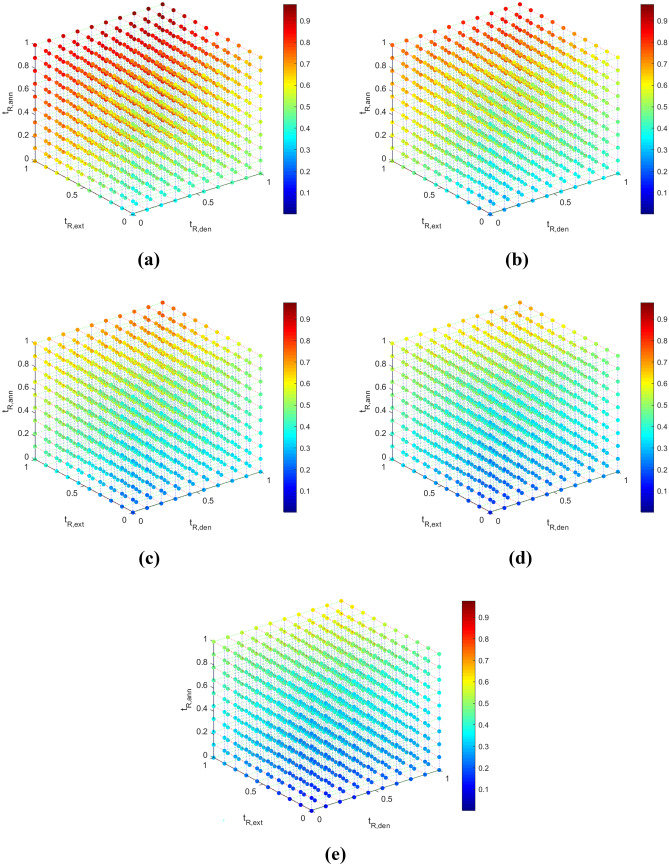
Visual representation of the $\Delta p$ (−) data (colorbar) for (**a**) $z_{w3}$ = 0, (**b**) $z_{w3}$ = $0.\bar{1}$, (**c**) $z_{w3}$ = $0.\bar{2}$, (**d**) $z_{w3}$ = $0.\bar{3}$, (**e**) $z_{w3}$ = $0.\bar{4}$

**Fig. 15:**
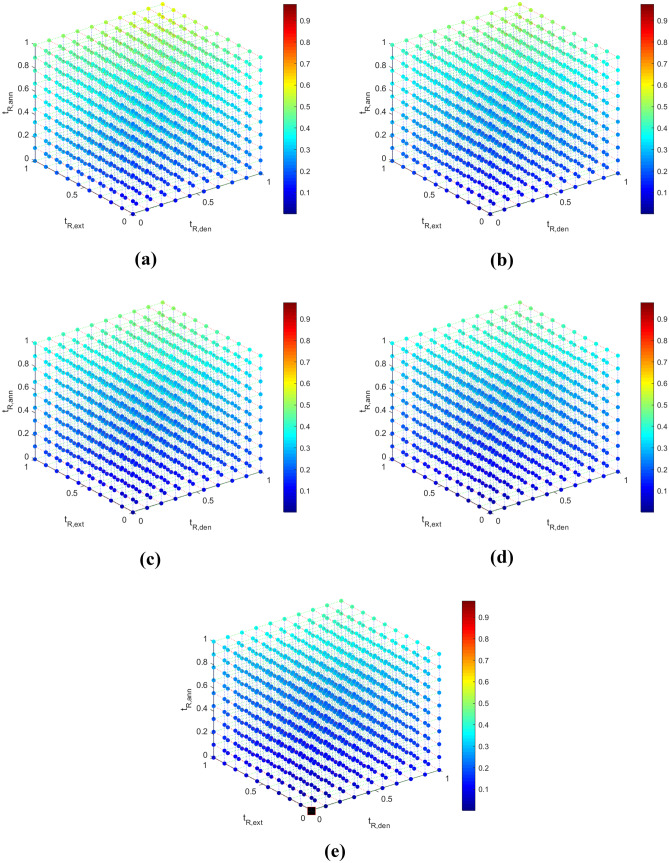
Visual representation of the $\Delta p$ (−) data (colorbar) for (**a**) $0.\bar{5}$, (**b**) $z_{w3}$ = $0.\bar{6}$, (**c**) $z_{w3}$ = $0.\bar{7}$, (**d**) $z_{w3}$ = $0.\bar{8}$, (**e**) $z_{w3}$ = 1

**Fig. 16:**
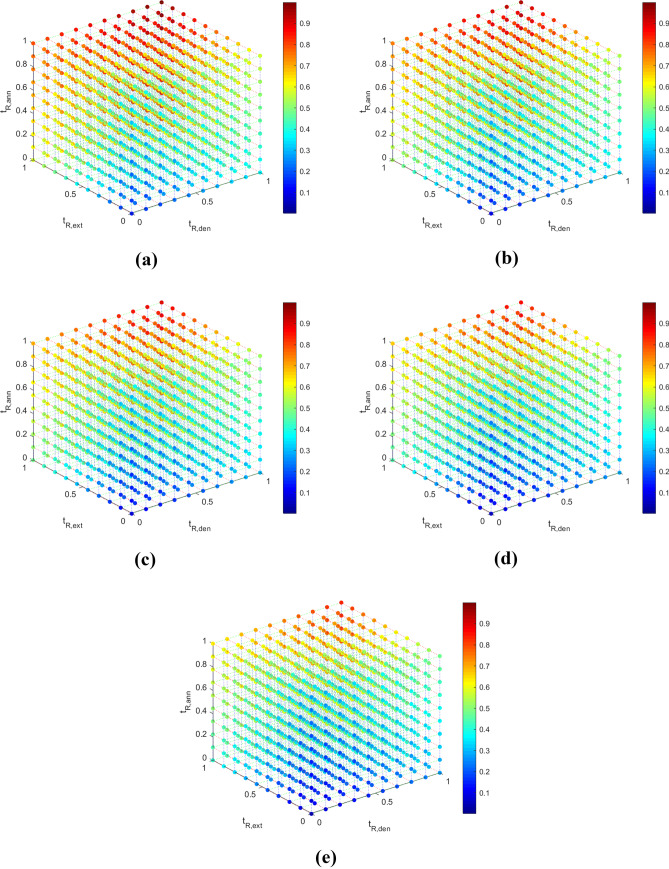
Visual representation of the $t_{R,tot}$ (−) data (colorbar) for (**a**) $z_{w3}$ = 0, (**b**) $z_{w3}$ = $0.\bar{1}$, (**c**) $z_{w3}$ = $0.\bar{2}$, (**d**) $z_{w3}$ = $0.\bar{3}$, (**e**) $z_{w3}$ = $0.\bar{4}$

**Fig. 17:**
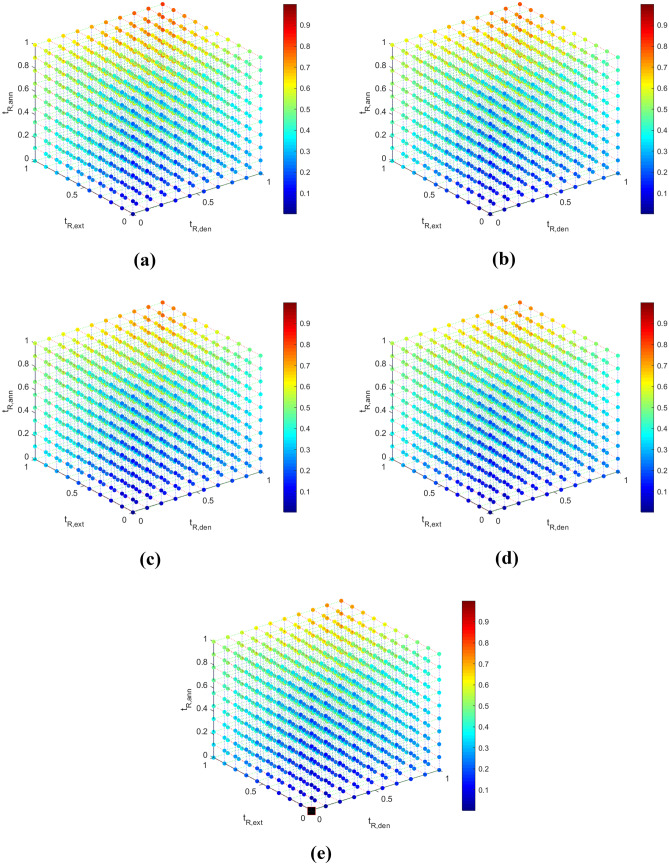
Visual representation of the $t_{R,tot}$ (−) data (colorbar) for (**a**) $0.\bar{5}$, (**b**) $z_{w3}$ = $0.\bar{6}$, (**c**) $z_{w3}$ = $0.\bar{7}$, (**d**) $z_{w3}$ = $0.\bar{8}$, (**e**) $z_{w3}$ = 1

**Fig. 18:**
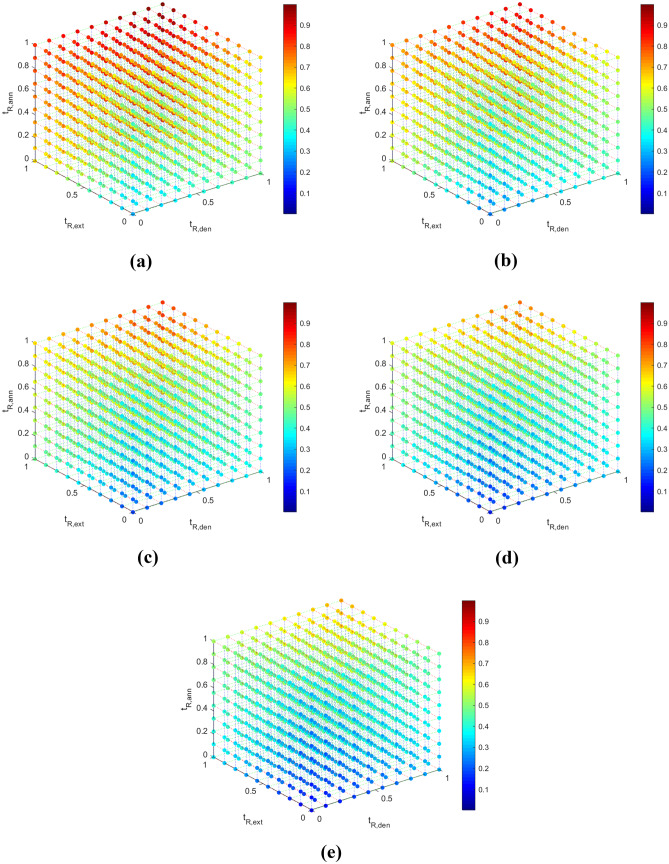
Visual representation of the $V_{S,tot}$ (−) data (colorbar) for (**a**) $z_{w3}$ = 0, (**b**) $z_{w3}$ = $0.\bar{1}$, (**c**) $z_{w3}$ = $0.\bar{2}$, (**d**) $z_{w3}$ = $0.\bar{3}$, (**e**) $z_{w3}$ = $0.\bar{4}$

**Fig. 19:**
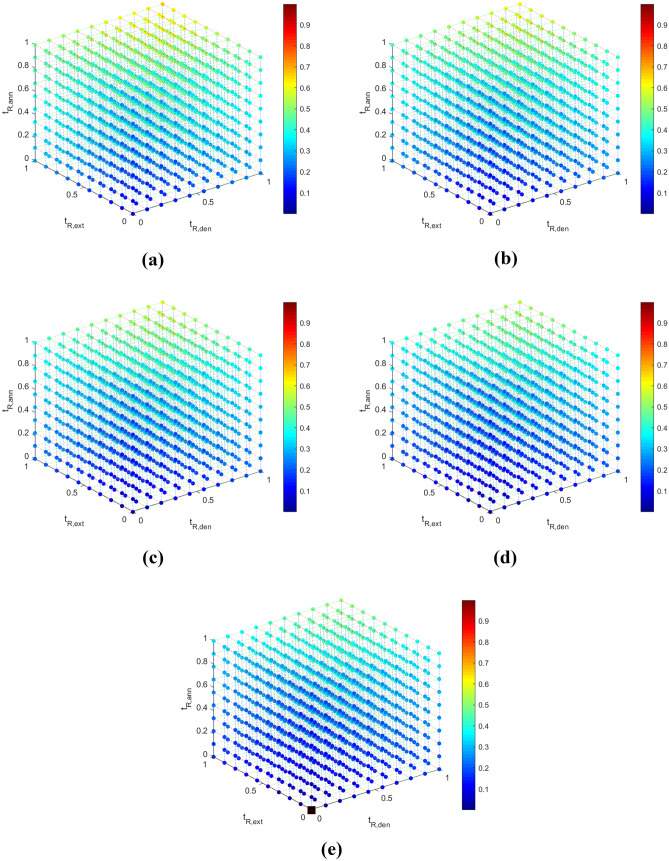
Visual representation of the $V_{S,tot}$ (−) data (colorbar) for (**a**) $0.\bar{5}$, (**b**) $z_{w3}$ = $0.\bar{6}$, (**c**) $z_{w3}$ = $0.\bar{7}$, (**d**) $z_{w3}$ = $0.\bar{8}$, **(e**) $z_{w3}$ = 1

## Data Availability

The datasets generated during and/or analysed during the current study are included in this published article and its supplementary information files.

## Appendix 1

### DoE points for the optimisation of the unit-cell

See Table 14

**Table 14:** DoE Points for the 4-design variable problem and obtained values

No	$t_{R, den}$	$t_{R, ext}$	$t_{R, ann}$	$z_{w3}$	$log_2 (-)$	$\Delta P (-)$	$t_{R, tot}$(-)	$V_{s, tot}$(-)
1	0.0000	0.0000	0.0000	0.0000	0.8894	0.3287	0.1506	0.2935
2	0.0000	0.0000	0.0000	1.0000	1.0000	0.0000	0.0000	0.0000
3	0.0000	0.0000	1.0000	0.0000	0.8541	0.5363	0.3630	0.4701
4	0.0000	0.0000	1.0000	1.0000	0.9782	0.2082	0.2124	0.1766
5	0.0000	1.0000	0.0000	0.0000	0.0194	0.6623	0.5753	0.6468
6	0.0000	1.0000	0.0000	1.0000	0.1183	0.1214	0.3186	0.1668
7	0.0000	1.0000	1.0000	0.0000	0.0004	0.8726	0.7877	0.8234
8	0.0000	1.0000	1.0000	1.0000	0.0956	0.3295	0.5309	0.3434
9	1.0000	0.0000	0.0000	0.0000	0.8766	0.4513	0.3630	0.4701
10	1.0000	0.0000	0.0000	1.0000	0.9993	0.1233	0.2124	0.1766
11	1.0000	0.0000	1.0000	0.0000	0.8633	0.6655	0.5753	0.6468
12	1.0000	0.0000	1.0000	1.0000	0.9658	0.3314	0.4247	0.3532
13	1.0000	1.0000	0.0000	0.0000	0.0190	0.7869	0.7877	0.8234
14	1.0000	1.0000	0.0000	1.0000	0.1141	0.2445	0.5309	0.3434
15	1.0000	1.0000	1.0000	0.0000	0.0000	1.0000	1.0000	1.0000
16	1.0000	1.0000	1.0000	1.0000	0.1030	0.4553	0.7432	0.5200
17	0.8741	0.3706	0.1399	0.2378	0.4546	0.4094	0.4480	0.4644
18	0.4895	0.2308	0.9860	0.9301	0.6458	0.3043	0.3927	0.3113
19	0.1259	0.5315	0.5524	0.6434	0.3441	0.2734	0.3561	0.2939
20	0.6224	0.9301	0.1189	0.2797	0.0875	0.4832	0.5916	0.5491
21	0.9580	0.7622	0.3147	0.0280	0.1191	0.7380	0.7315	0.7639
22	0.6853	0.2028	0.5734	0.5175	0.6548	0.3278	0.3840	0.3617
23	0.6503	0.0000	0.4056	0.4476	0.9374	0.2719	0.2790	0.2987
24	0.5524	0.6643	0.9650	0.6783	0.2520	0.4231	0.5742	0.4590
25	0.9650	0.6573	0.5664	0.3357	0.2274	0.5269	0.6416	0.5873
26	0.5105	0.8671	0.6224	0.8112	0.1431	0.3400	0.5407	0.3923
27	0.8951	0.6154	0.8392	0.7413	0.2840	0.4146	0.5947	0.4698
28	0.9441	0.1399	0.1678	0.1329	0.6936	0.4269	0.4057	0.4645
29	0.8811	0.1049	0.8741	0.7832	0.7930	0.3372	0.4243	0.3657
30	0.5594	0.9510	0.8252	0.8462	0.1118	0.3912	0.6168	0.4425
31	0.3916	0.8112	0.1329	0.9021	0.1917	0.1947	0.3812	0.2509
32	0.5664	0.7832	0.1538	0.7972	0.2003	0.2411	0.4274	0.3076
33	0.0070	0.4126	0.7552	0.8811	0.4535	0.2289	0.3047	0.2269
34	0.9371	0.1748	0.4476	0.5664	0.6867	0.3142	0.3941	0.3642
35	0.1888	0.5245	0.9580	0.2308	0.3065	0.5384	0.5372	0.5348
36	0.2517	0.6853	0.3846	0.5105	0.2305	0.3233	0.4232	0.3635
37	0.4336	0.2797	0.0000	1.0000	0.6165	0.0875	0.1812	0.1232
38	0.9510	0.2517	0.2378	0.7902	0.6245	0.2330	0.3520	0.2920
39	0.0350	0.2937	0.9231	0.0070	0.4811	0.6285	0.4762	0.5617
40	0.1678	0.2448	0.4965	0.3427	0.5768	0.3173	0.3029	0.3244
41	0.1329	0.9371	0.7762	0.8252	0.1198	0.3322	0.5143	0.3618
42	0.6434	0.9650	0.5594	0.8951	0.1138	0.3361	0.5760	0.3997
43	0.9790	0.7692	0.8951	0.9161	0.2014	0.4180	0.6525	0.4785
44	0.0000	0.5594	0.3357	0.9720	0.3453	0.1428	0.2523	0.1583
45	0.7063	0.0629	0.0140	0.4336	0.8533	0.2138	0.2324	0.2586
46	0.4196	0.9021	0.2587	0.1049	0.0675	0.6213	0.6296	0.6444
47	0.8112	0.8322	0.6364	0.6643	0.1577	0.4167	0.6186	0.4852
48	0.9720	0.0839	0.0909	0.2937	0.8009	0.3178	0.3370	0.3707
49	0.1538	0.8531	0.1958	0.7343	0.1596	0.2261	0.3812	0.2735
50	0.3007	0.4196	0.3566	0.6853	0.4422	0.2259	0.3077	0.2557
51	0.7902	0.0559	0.1818	0.3497	0.8316	0.2864	0.2970	0.3277
52	0.7622	0.3357	0.5245	0.1888	0.4679	0.5000	0.5052	0.5264
53	0.6713	0.0420	0.8881	0.6503	0.8876	0.3315	0.3752	0.3465
54	0.6993	0.3497	0.4266	0.7483	0.5088	0.2638	0.3758	0.3090
55	0.0490	0.0070	0.6713	0.9510	0.9634	0.1534	0.1587	0.1358
56	0.2727	0.0909	0.5804	0.0839	0.7392	0.4481	0.3444	0.4283
57	0.6364	0.9091	0.3427	0.1748	0.0763	0.6048	0.6691	0.6500
58	0.8042	0.4336	0.6573	0.1399	0.3706	0.5917	0.5952	0.6131
59	0.2028	0.3636	0.3007	0.3846	0.4714	0.2885	0.3041	0.3104
60	0.7832	0.1469	0.9720	0.7133	0.7407	0.3661	0.4455	0.3884
61	0.7692	0.3427	0.8531	0.9930	0.5196	0.3154	0.4543	0.3449
62	0.9231	0.5524	0.2238	0.1818	0.2879	0.5163	0.5624	0.5694
63	0.1748	0.5385	0.3986	0.8392	0.3528	0.2006	0.3103	0.2256
64	0.8252	0.4476	0.8112	0.8601	0.4235	0.3501	0.5040	0.3921
65	0.3846	0.7552	0.1259	0.5524	0.1978	0.2826	0.4130	0.3416
66	0.7552	0.5664	0.9161	0.5944	0.3098	0.4470	0.5866	0.4914
67	0.3636	0.9580	0.6783	0.0350	0.0233	0.7935	0.7612	0.7841
68	0.0769	0.5035	0.6294	0.8042	0.3748	0.2392	0.3311	0.2509
69	0.4825	0.4266	0.7832	0.1469	0.3731	0.5711	0.5487	0.5724
70	0.0839	0.2378	0.2937	0.4755	0.6129	0.2149	0.2158	0.2267
71	0.1049	0.7133	0.0350	0.8741	0.2500	0.1323	0.2712	0.1724
72	0.6573	0.3776	0.3217	0.2028	0.4269	0.4424	0.4524	0.4773
73	0.6084	0.6364	0.0979	0.5035	0.2655	0.3019	0.4221	0.3673
74	0.6294	0.2587	0.6503	0.3287	0.5706	0.4161	0.4415	0.4421
75	0.1958	0.0210	0.4615	0.7692	0.9302	0.1575	0.1647	0.1585
76	0.2937	0.8042	0.8601	0.0210	0.0885	0.7907	0.7271	0.7632
77	0.8392	0.6084	0.7692	0.3706	0.2584	0.5266	0.6315	0.5727
78	0.5385	0.0699	0.4685	0.3147	0.8093	0.3312	0.3159	0.3495
79	0.5245	0.9441	0.5455	0.4965	0.0954	0.4429	0.6097	0.5047
80	0.6643	0.1678	0.9510	0.2168	0.6529	0.5150	0.5027	0.5226
81	0.6014	0.6783	0.8671	0.9231	0.2530	0.3521	0.5362	0.3894
82	0.5734	0.8182	0.2517	0.3217	0.1412	0.4506	0.5551	0.5114
83	0.6923	0.8741	0.1049	0.0699	0.0814	0.6497	0.6584	0.6835
84	0.6154	0.4755	0.2727	0.7552	0.3952	0.2382	0.3665	0.2896
85	0.8322	0.8462	0.8182	0.1189	0.0889	0.7608	0.8079	0.7882
86	0.2308	0.2238	0.0769	0.2098	0.6106	0.2971	0.2484	0.3108
87	0.8182	0.1888	0.3636	0.9650	0.6972	0.2048	0.3139	0.2461
88	0.0699	0.9231	0.1748	0.0979	0.0597	0.5727	0.5490	0.5799
89	0.3077	0.8881	0.0629	0.8182	0.1549	0.2001	0.3848	0.2596
90	0.5455	0.4615	0.1888	0.4825	0.3877	0.2883	0.3700	0.3394
91	0.5315	0.3566	0.0490	0.5594	0.4944	0.2142	0.2876	0.2640
92	0.9091	0.1958	0.7972	0.0140	0.5904	0.6647	0.5911	0.6550
93	0.4476	0.8601	0.3776	0.5804	0.1340	0.3504	0.5110	0.4099
94	0.3706	0.9860	0.0420	0.3916	0.0745	0.3778	0.5106	0.4463
95	0.0140	1.0000	0.4755	0.2517	0.0470	0.5191	0.5736	0.5404
96	0.0559	0.5874	0.2797	0.4615	0.2900	0.2784	0.3342	0.3060
97	0.4266	0.0979	0.2098	0.0420	0.7303	0.4338	0.3137	0.4154
98	0.4965	0.7413	0.4196	0.8322	0.2186	0.2744	0.4505	0.3251
99	0.8601	0.4406	0.4895	0.8531	0.4329	0.2881	0.4415	0.3417
100	0.3217	0.0769	0.9441	0.9860	0.8537	0.2477	0.2944	0.2386
101	1.0000	0.1119	0.3077	0.9441	0.8062	0.2088	0.3176	0.2587
102	0.0210	0.5105	0.9021	0.5734	0.3433	0.3504	0.4117	0.3538
103	0.1608	0.8392	0.4545	0.0559	0.0862	0.6613	0.6115	0.6511
104	0.4685	0.9790	0.2448	0.4685	0.0815	0.3924	0.5528	0.4618
105	0.0979	0.6503	0.9091	0.1678	0.2057	0.5948	0.5756	0.5821
106	0.3986	0.4895	0.1119	0.7622	0.3952	0.1787	0.2901	0.2239
107	0.5175	0.3287	0.6084	0.6154	0.5222	0.3085	0.3857	0.3384
108	0.2448	0.5804	0.0839	0.0909	0.2505	0.4773	0.4303	0.4898
109	0.2587	0.6713	0.7133	0.9371	0.2604	0.2741	0.4270	0.2973
110	0.1119	0.1818	0.0070	0.6713	0.7117	0.0963	0.1145	0.1159
111	0.7133	0.7483	0.7413	0.4406	0.1788	0.4984	0.6339	0.5501
112	0.4126	0.1538	0.7622	0.1119	0.6666	0.5035	0.4304	0.4906
113	0.4406	0.1329	0.6643	0.4266	0.7373	0.3321	0.3405	0.3455
114	0.9930	0.2657	0.0699	0.4196	0.5858	0.3051	0.3808	0.3726
115	0.3357	0.3217	0.8462	0.0769	0.4540	0.5876	0.5108	0.5655
116	0.8462	0.6014	0.2867	0.6084	0.2832	0.3282	0.4820	0.3994
117	0.0909	0.9720	0.2657	0.2657	0.0655	0.4628	0.5302	0.5008
118	0.4615	0.4545	0.2168	0.2867	0.3721	0.3675	0.3966	0.4055
119	0.2657	0.6224	1.0000	0.9580	0.2893	0.3242	0.4714	0.3362
120	0.7343	0.4965	0.6923	0.3986	0.3365	0.4569	0.5458	0.5011
121	0.9021	0.3916	0.8322	0.5455	0.4484	0.4332	0.5470	0.4797
122	0.8531	0.2727	0.7203	0.7203	0.5888	0.3391	0.4483	0.3795
123	0.5944	0.4056	0.6853	0.0000	0.3682	0.6839	0.5946	0.6628
124	0.3566	0.1189	0.0280	0.4056	0.7661	0.1937	0.1859	0.2215
125	0.3497	0.2098	0.5315	0.5874	0.6534	0.2598	0.2965	0.2773
126	0.0629	0.2867	0.6154	0.7273	0.5708	0.2203	0.2622	0.2226
127	0.2867	0.4685	0.6993	0.3776	0.3576	0.4066	0.4464	0.4246
128	0.5804	0.5175	0.3497	0.9091	0.3725	0.2235	0.3715	0.2692
129	0.4056	0.7762	0.5944	0.1958	0.1377	0.5775	0.6142	0.6010
130	0.7413	0.0140	0.9371	0.8671	0.9471	0.3067	0.3707	0.3199
131	0.8881	0.8811	0.1608	0.3077	0.1092	0.4968	0.6301	0.5748
132	0.7273	0.5944	0.0559	0.1538	0.2471	0.4843	0.5106	0.5342
133	0.4545	0.1608	0.1469	0.4895	0.7130	0.2115	0.2338	0.2442
134	0.7762	0.3007	0.5175	0.6224	0.5547	0.3151	0.4107	0.3602
135	0.1818	0.6923	0.7343	0.1259	0.1709	0.6123	0.5875	0.6055
136	0.9161	0.3846	0.3916	0.6364	0.4716	0.3161	0.4405	0.3765
137	0.4755	0.2168	0.4126	0.6923	0.6533	0.2261	0.2872	0.2539
138	0.8671	0.8252	0.4406	0.5315	0.1506	0.4277	0.6109	0.5057
139	0.7483	0.9930	0.7063	0.1608	0.0353	0.7281	0.8085	0.7688
140	0.3776	0.7063	0.5385	0.9790	0.2366	0.2481	0.4218	0.2841
141	0.3427	0.7902	0.8811	0.5245	0.1650	0.4514	0.5821	0.4857
142	0.7203	0.6993	0.9790	0.2727	0.1930	0.6278	0.7096	0.6592
143	0.6783	0.5455	0.6434	0.6993	0.3343	0.3479	0.4894	0.3945
144	0.3287	0.0350	0.5874	0.8881	0.9165	0.1829	0.2141	0.1855
145	0.2238	0.7972	0.9301	0.3566	0.1475	0.5280	0.6079	0.5467
146	0.0420	0.5734	0.9930	0.3636	0.2871	0.4658	0.4985	0.4656
147	0.9860	0.7343	0.7902	0.4545	0.1902	0.5357	0.6941	0.5979
148	0.5874	0.3147	0.8042	0.4126	0.5094	0.4198	0.4696	0.4450
149	0.2378	0.1259	0.7273	0.3007	0.7266	0.3667	0.3307	0.3621
150	0.1469	0.8951	0.3706	0.5385	0.1235	0.3338	0.4662	0.3790
151	0.9301	0.7273	0.4825	0.7762	0.2206	0.3522	0.5588	0.4250
152	0.3147	0.6294	0.5105	0.2238	0.2272	0.4882	0.5114	0.5115
153	0.2168	0.7203	0.3287	0.7063	0.2234	0.2493	0.3825	0.2903
154	0.2797	0.9161	0.6014	0.6294	0.1183	0.3684	0.3825	0.2903
155	0.1189	0.3077	0.5035	0.0629	0.4726	0.4934	0.3825	0.2903
156	0.7972	0.0280	0.7483	0.2587	0.8697	0.4360	0.3825	0.2903
157	0.0280	0.6434	0.0210	0.0490	0.2105	0.4958	0.3825	0.2903
158	0.1399	0.3986	0.2028	0.6014	0.4594	0.1927	0.2454	0.2188
159	0.2098	0.0490	0.2308	0.6573	0.8836	0.1370	0.1393	0.1488
160	0.5035	0.4825	0.4336	0.2448	0.3431	0.4481	0.4728	0.4789

## Appendix 2

### Kinetics

In the denaturation zone, the double-stranded DNA molecules, $S{_1}S{_2}$, dissociate into two single strands, $S{_1}$ and $S{_2}$ (Reaction 14):

14 $$\begin{aligned} S_1S_2 \overset{{k_D^+}} {\underset{k_{D^-}}\longleftrightarrow} S_1 + S_2 \end{aligned}$$

where $k_D$ is the denaturation constant for melting of D at melting temperature; $k_{-D}$ is the denaturation constant for binding of S at melting temperature; $k_E$ is the enzyme inactivation constant. The arrow symbols “$\leftarrow$” and “$\rightarrow$” are used to denote net forward and backward reactions. The high melting temperature causes thermal denaturation of the enzyme responsible for the DNA amplification.

In annealing zone, the single-stranded primer molecules, $P{_1}$ and $P{_2}$, bind to $S{_2}$ and $S{_1}$ respectively, and form the single-stranded template–primer complexes, $P{_1}S{_2}$ and $S{_1}P{_2}$ (Reactions 15 and 16):

15 $$\begin{aligned} S_1 + P_2 \overset{k_A^+}{\underset{k_{A^-}} \longleftrightarrow} S_1P_2 \end{aligned}$$

16 $$\begin{aligned} S_2 + P_1 \overset{k_A^+}{\underset{k_{A^-}} \longleftrightarrow} P_1S_2 \end{aligned}$$

where $k_{A}^+$ is the annealing coefficient of $P{_1}$ and $P{_2}$ to $S{_2}$ and $S{_1}$ respectively,; $k_{A}^-$ is the dissociation coefficient of $P{_1}S{_2}$ and $S{_1}P{_2}$.

In extension zone, the polymerase enzyme binds to $P{_1}S{_2}$ and $S{_1}P{_2}$ to form the single-stranded template–primer–enzyme complexes. Then these complexes dissociate into the enzyme and the DNA molecules at the beginning of the subsequent denaturation step (Reactions 17 and 18):

17 $$\begin{aligned} S_1P_2 \overset{k_E}\longrightarrow S_1S_2 \end{aligned}$$

18 $$\begin{aligned} P_1S_2 \overset{k_E}\longrightarrow S_1S_2 \end{aligned}$$

where $k_E$ is the addition constant of the enzyme to $P{_1}S{_2}$ and $S{_1}P{_2}$.

The temperature dependence of the various rate constants mentioned earlier, $k_D^+, k_D^-, k_E, k_A^+$ and $k_A^-$, are given by Eqs. 19–23, as demonstrated also in the work of Papadopoulos et al. (2015):

19 $$\begin{aligned} k_{D}^+ (T) = 0.5 \cdot k_{o}^+ \cdot \left( 1+tanh \left( \frac{T -361.15}{5}\right) \right) \end{aligned}$$

20 $$\begin{aligned} k_{D}^- (T) = 0.5 \cdot k_{o}^- \cdot \left( 1+tanh \left( \frac{348.15 - T}{5}\right) \right) \end{aligned}$$

21 $$\begin{aligned} k_{A}^+ (T) = 0.5 \cdot k_{1}^+ \cdot \left( 1+tanh \left( \frac{335.65 - T}{5}\right) \right) \end{aligned}$$

22 $$\begin{aligned} k_{A}^- (T) = 0.5 \cdot k_{1}^- \cdot \left( 1+tanh \left( \frac{T - 339.15}{5}\right) \right) \end{aligned}$$

23 $$\begin{aligned} k_{E} (T) = k_{2}\cdot exp \left( - \left( \frac{T - 345.15}{5}\right) ^2\right) \end{aligned}$$

where *T* is the temperature in *K*, and the $k_o^+$, $k_o^-$, $k_1^+$, $k_1^-$ and $k_2$ constants are presented in Table 15. The reaction rates are given by Eqs. 24–30.

24 $$\begin{aligned} R_1 = -k_D^+ C_1 + k_D^- C_2C_3 + k_E C_6 C_7 \end{aligned}$$

25 $$\begin{aligned} R_2 = k_D^+ C_1 - k_D^- C_2C_3 - k_A^+ C_2C_5 + k_A^- C_7 \end{aligned}$$

26 $$\begin{aligned} R_3 = k_D^+ C_1 - k_D^- C_2C_3 - k_A^+ C_3C_4 + k_A^- C_6 \end{aligned}$$

27 $$\begin{aligned} R_4 = - k_A^+ C_3C_4 + k_A^- C_6 \end{aligned}$$

28 $$\begin{aligned} R_5 = - k_A^+ C_5C_2 + k_A^- C_7 \end{aligned}$$

29 $$\begin{aligned} R_6 = k_A^+ C_4C_3 - (k_A^- +k_E)C_6 \end{aligned}$$

30 $$\begin{aligned} R_7 = k_A^+ C_2C_5 - (k_A^- +k_E)C_7 \end{aligned}$$

**Table 15:** Values of constants in reaction rate constants (Papadopoulos et al. 2015)

Parameter	Values	Description
$k_{o}^+$	12.5[1/*s*]	Constant parameter in reaction rate constant $k_{D}^+$
$k_{o}^-$	$10^3 [m^3/(mol \cdot s)]$	Constant parameter in reaction rate constant $k_{D}^-$
$k_{1}^+$	$5 \cdot 10^3 [m^3/(mol \cdot s)]$	Constant parameter in reaction rate constant $k_{A}^+$
$k_{1}^-$	$10^{-4} [1/s]$	Constant parameter in reaction rate constant $k_{A}^-$
$k_{2}$	0.32[1/*s*]	Constant parameter in reaction rate constant $k_{E}$

## Appendix 3

### Calculation of power consumption

Using the Joule Heating model, the power consumption for the heater in the denaturation regime for the design case presented in the work of Papadopoulos et al. (2015) is calculated as follows:

31 $$\begin{aligned} A_{heater} =W_{heater} \cdot H_{heater} = 2\cdot 10^{-9}m^2 \end{aligned}$$

32 $$\begin{aligned} J_{normal}=I_{den}/A_{heater}=\frac{0.2345 [A]}{2\cdot 10^{-9}[m^2]} =1.17\cdot 10^{8}[A/m^2] \end{aligned}$$

33 $$\begin{aligned} \varrho (T)_{den}= \varrho _{293K}[1+\alpha (T_{den}-T_{ref})] =2.16636 \cdot 10^{-8} [\Omega m] \end{aligned}$$

34 $$\begin{aligned} L_{den,heater} = 8\cdot 0.0071 [m]+9\cdot 200\cdot 10^{-6} [m] =0.0586[m] \end{aligned}$$

35 $$\begin{aligned} \mathcal {R}_{den}&=\varrho _{DEN, 293K}\frac{L_{den,heater}}{A_{heater}}\\& =2.16636 \cdot 10^{-8}[\Omega m]\frac{ 0.0586[m]}{2\cdot 10^{-9}[m^2]} \\&= 0.63474 [\Omega ] \end{aligned}$$

36 $$\begin{aligned} P_{den}= \mathcal {R}_{den} I_{den}^2 =0.63474 [\Omega ] \cdot (0.2345 [A])^2 = 0.03490 [W] \end{aligned}$$

where $\alpha$ : the coefficient of thermal expansion of copper (0.0386 $K^{-1}$), $\varrho _{293K}$ : the reference resistivity of copper at 293 K (1.68 $\cdot 10^{-8}$ $\Omega m$), $\varrho (T)_{den}$ : the resistivity of the copper-wire heater at denaturation zone, $T_{ref}:293.15 K$, $T_{den}:368.15 K$, $\mathcal {R}$: the resistance ($\Omega$) and P the power consumption (W). The width ($W_{heater}$), height ($H_{heater}$) and length ($L_{den,heater}$) of the copper wires used in the simulations is $10^{-4}$ m, $2\cdot 10^{-5}$ m and 0.0586 m respectively, while the value of $I_{den}$ is found to be equal to 0.2345 A by trial and error. The copper wire is bent nine times, resulting to eight straight parts ($L_{den,straight}$=0.0071 m) covering the bottom of the heater in a serpentine shape (see Fig. 2). The power consumption of the heaters of extension and annealing zones are calculated in the same way, and are equal to 0.02367 and 0.01201 W respectively.

## Appendix 4

### Concentrations of all PCR products for 10 cycles

See Tables 16 and 17

**Table 16:** Concentrations of all PCR products for 10 cycles for Design 4 (see Table 10)

Design	$C_1$	$C_2$	$C_3$	$C_4$	$C_5$	$C_6$	$C_7$
Design 4 (see Table 10)	($mol/m^3$)	($mol/m^3$)	($mol/m^3$)	($mol/m^3$)	($mol/m^3$)	($mol/m^3$)	($mol/m^3$)
Start	5.7100 ⋅ 10^−9^	0.0000	0.0000	3.0000 ⋅ 10^−4^	3.0000 ⋅ 10^−4^	0.0000	0.0000
Cycle 1	9.7358 ⋅ 10^−9^	5.0622 ⋅ 10^−10^	5.0622 ⋅ 10^−10^	2.9999 ⋅ 10^−4^	2.9999 ⋅ 10^−4^	6.7201 ⋅ 10^−10^	6.7201 ⋅ 10^−10^
Cycle 2	1.8816 ⋅ 10^−8^	9.8893 ⋅ 10^−10^	9.8893 ⋅ 10^−10^	2.9998 ⋅ 10^−4^	2.9998 ⋅ 10^−4^	1.2395 ⋅ 10^−9^	1.2395 ⋅ 10^−9^
Cycle 3	3.6261 ⋅ 10^−8^	1.9046 ⋅ 10^−9^	1.9046 ⋅ 10^−9^	2.9996 ⋅ 10^−4^	2.9996 ⋅ 10^−4^	2.3936 ⋅ 10^−9^	2.3936 ⋅ 10^−9^
Cycle 4	6.9871 ⋅ 10^−8^	3.6683 ⋅ 10^−9^	3.6683 ⋅ 10^−9^	2.9992 ⋅ 10^−4^	2.9992 ⋅ 10^−4^	4.6117 ⋅ 10^−9^	4.6117 ⋅ 10^−9^
Cycle 5	1.3455 ⋅ 10^−7^	7.0563 ⋅ 10^−9^	7.0563 ⋅ 10^−9^	2.9984 ⋅ 10^−4^	2.9984 ⋅ 10^−4^	8.8753 ⋅ 10^−9^	8.8753 ⋅ 10^−9^
Cycle 6	2.5892 ⋅ 10^−7^	1.3545 ⋅ 10^−8^	1.3545 ⋅ 10^−8^	2.9969 ⋅ 10^−4^	2.9969 ⋅ 10^−4^	1.7037 ⋅ 10^−8^	1.7037 ⋅ 10^−8^
Cycle 7	4.9806 ⋅ 10^−7^	2.5982 ⋅ 10^−8^	2.5982 ⋅ 10^−8^	2.9941 ⋅ 10^−4^	2.9941 ⋅ 10^−4^	3.2591 ⋅ 10^−8^	3.2591 ⋅ 10^−8^
Cycle 8	9.5834 ⋅ 10^−7^	4.9859 ⋅ 10^−8^	4.9859 ⋅ 10^−8^	2.9887 ⋅ 10^−4^	2.9887 ⋅ 10^−4^	6.2271 ⋅ 10^−8^	6.2271 ⋅ 10^−8^
Cycle 9	1.8427 ⋅ 10^−6^	9.5281 ⋅ 10^−8^	9.5281 ⋅ 10^−8^	2.9783 ⋅ 10^−4^	2.9783 ⋅ 10^−4^	1.1961 ⋅ 10^−7^	1.1961 ⋅ 10^−7^
Cycle 10	3.5361 ⋅ 10^−6^	1.8177 ⋅ 10^−7^	1.8177 ⋅ 10^−7^	2.9583 ⋅ 10^−4^	2.9583 ⋅ 10^−4^	2.2951 ⋅ 10^−7^	2.2951 ⋅ 10^−7^

**Table 17:** Concentrations of all PCR products for 10 cycles for Design 2 (see Table 10)

Design	$C_1$	$C_2$	$C_3$	$C_4$	$C_5$	$C_6$	$C_7$
Design 2 (see Table 10)	($mol/m^3$)	($mol/m^3$)	($mol/m^3$)	($mol/m^3$)	($mol/m^3$)	($mol/m^3$)	($mol/m^3$)
Start	5.71 ⋅ 10^−9^	0	0	3.0000 ⋅ 10^−4^	3.0000 ⋅ 10^−4^	0	0
Cycle 1	9.066 ⋅ 10^−9^	8.0298 ⋅ 10^−10^	8.0298 ⋅ 10^−10^	2.9998 ⋅ 10^−4^	2.9998 ⋅ 10^−4^	8.1024 ⋅ 10^−10^	8.1024 ⋅ 10^−10^
Cycle 2	1.7119 ⋅ 10^−8^	1.5355 ⋅ 10^−9^	1.5355 ⋅ 10^−9^	2.9998 ⋅ 10^−4^	2.9998 ⋅ 10^−4^	1.4611 ⋅ 10^−9^	1.4611 ⋅ 10^−9^
Cycle 3	3.2232 ⋅ 10^−8^	2.8895 ⋅ 10^−9^	2.8895 ⋅ 10^−9^	2.9997 ⋅ 10^−4^	2.9997 ⋅ 10^−4^	2.7564 ⋅ 10^−9^	2.7564 ⋅ 10^−9^
Cycle 4	6.0689 ⋅ 10^−8^	5.4356 ⋅ 10^−9^	5.4356 ⋅ 10^−9^	2.9995 ⋅ 10^−4^	2.9995 ⋅ 10^−4^	5.1896 ⋅ 10^−9^	5.1896 ⋅ 10^−9^
Cycle 5	1.1422 ⋅ 10^−7^	1.0193 ⋅ 10^−8^	1.0193 ⋅ 10^−8^	2.9992 ⋅ 10^−4^	2.9992 ⋅ 10^−4^	9.7721 ⋅ 10^−9^	9.7721 ⋅ 10^−9^
Cycle 6	2.1476 ⋅ 10^−7^	1.9007 ⋅ 10^−8^	1.9007 ⋅ 10^−8^	2.9986 ⋅ 10^−4^	2.9986 ⋅ 10^−4^	1.8402 ⋅ 10^−8^	1.8402 ⋅ 10^−8^
Cycle 7	4.0357 ⋅ 10^−7^	3.534 ⋅ 10^−8^	3.534 ⋅ 10^−8^	2.9975 ⋅ 10^−4^	2.9975 ⋅ 10^−4^	3.4602 ⋅ 10^−8^	3.4602 ⋅ 10^−8^
Cycle 8	7.5864 ⋅ 10^−7^	6.5717 ⋅ 10^−8^	6.5717 ⋅ 10^−8^	2.9935 ⋅ 10^−4^	2.9935 ⋅ 10^−4^	6.5075 ⋅ 10^−8^	6.5075 ⋅ 10^−8^
Cycle 9	1.4256 ⋅ 10^−6^	1.2203 ⋅ 10^−7^	1.2203 ⋅ 10^−7^	2.9856 ⋅ 10^−4^	2.9856 ⋅ 10^−4^	1.2279 ⋅ 10^−7^	1.2279 ⋅ 10^−7^
Cycle 10	2.6751 ⋅ 10^−6^	2.2813 ⋅ 10^−7^	2.2813 ⋅ 10^−7^	2.9703 ⋅ 10^−4^	2.9703 ⋅ 10^−4^	2.3102 ⋅ 10^−7^	2.3102 ⋅ 10^−7^

## Appendix 5

### Visual representation of the four objective functions of interest; $log_2 \frac{[DNA]}{[DNA]_{o}}$, $\Delta p$, $t_{R,tot}$ and $V_{S,tot}$

#### Visual representation of the $log_2 \frac{[DNA]}{[DNA]_{o}}$ (−) objective

See Figs. 12 and 13

#### Visual representation of the $\Delta p$ (−) objective

See Figs. 14 and 15

#### Visual representation of the $t_{R,tot}$ (−) objective

See Figs. 16 and 17

#### Visual representation of the $V_{S,tot}$ (−) objective

See Figs. 18 and 19

## Appendix 6

### Scaling of the objective functions

All values of the objective functions are scaled between 0−1 for the purposes of multi-objective optimisation. Furthermore, just for the first objective, the values of $-log_2 \frac{[DNA]}{[DNA]_{o}}$ are scaled between 0−1, in order for all optimisation studies to be minimisation problems. Equation 37 is used to scale all values, while Table 18 presents two cases of scaling values of objectives.

37 $$\begin{aligned} V_{4}=\frac{V_1 -V_3}{V_2 -V_3}={\left\{ \begin{array}{ll} \frac{(-0.617) -(-0.772)}{(-0.597) -(-0.772)}, &{} \text {Example 1}\\ \frac{(88.437) -(55.436)}{(140.622) -(55.436)},&{} \text {Example 2} \end{array}\right. } \end{aligned}$$

**Table 18:** Calculations for scaling the values of objective functions

Example	Objective	Starting	max($V_1$),	min($V_1$),	Scaled
		Value, $V_1$	$V_2$	$V_3$	value, $V_4$
1	$-log_2 \frac{[DNA]}{[DNA]_{o}}$	−0.617 (−)	−0.597 (−)	−0.772 (−)	0.889 (−)
2	$\Delta p$	88.437 (Pa)	140.622 (Pa)	55.436 (Pa)	0.329 (−)

## Nomenclature

$\alpha$: Coefficient of thermal expansion [$K^{-1}$]
$\Delta P$: Pressure Drop [Pa]
$$\epsilon$$: Surface emissivity [-]
$$\lambda$$: PCR efficiency [−]
$$\mu$$: Dynamic viscosity [$$Pa\cdot s$$]
$$\rho$$: Density [$$kg/m^3$$]
$\varrho (T)$: Resistivity of copper wire [$\Omega m$]
$\sigma$: Stefan–Boltzmann constant ($5.670373\cdot 10^{-8}$ [$W/(m^{2}K^{4})$])
*A*: Cross-sectional area [$m^2$]
$C_p$: Heat capacity at constant pressure [J/K]
$C_k$: Concentration of $k^{th}$ species taking part in PCR [$mol/m^3$]
$D_k$: Diffusion Coefficient of $k^{th}$ species taking part in PCR [$m^2/s$]
$$D_h$$: Hydraulic diameter [m]
*h*: Heat transfer coefficient [$W/(m^2\cdot K)$]
*H*: Height [m]
$$\mathbf{I}$$: Identity matrix [-]
*I*: Current [A]
*J*: Diffusion flux [$$mol/(s \cdot m^2)$$]
$J_{normal}$: Normal current density [$A/(m^2)$]
$\mathbf{F}$: External forces applied in a volume of fluid [N]
$k_{i}^j$: Reaction rate constant i, where i = {D, A, E}, j = {+, -} [$mol/(s \cdot m^3)$]
*k*: Thermal conductivity [$$W/(m\cdot K)$$]
*L*: Length [m]
*N*: Number of PCR cycles [-]
$N_{DVARS}$: Number of design variables [-]
$\mathcal {N}$: Number of base pairs [-]
*P*: Power consumption [W]
*p*: Pressure [Pa]
$P_1$: Single - stranded primer molecule [-]
$P_2$: Single - stranded primer molecule [-]
$P_1S_2$: Single-stranded template–primer complex [-]
$Q_{heater,j}$: Heat generation rate of the $j^{th}$ heater, where j = {1,2,3} [$W/m^3$]
$Q_{nat.conv.,i}$: Heat flux due to natural convection of the $i^{th}$ material, where i = {Kapton, PE, PDMS, Copper} [$W/m^3$]
$Q_{rad,i}$: Heat flux due to thermal radiation of the $i^{th}$ material, where i = {Kapton, PE, PDMS, Copper} [$W/m^3$]
$$Q_{vol}$$: Volumetric flowrate [$$m^3/s$$]
$$\mathcal {R}$$: Resistance [$$\Omega$$]
*Re*: Reynolds number [-]
$R_{k}$: Net reaction rate defined by PCR kinetics [$mol/(s \cdot m^3)$]
$R_{zone}$: Radius of the circular part of the microchannel [$\mu m$]
$S_1S_2$: Double - stranded DNA molecule [-]
$S_1$: Single - stranded DNA molecule [-]
$S_2$: Single - stranded DNA molecule [-]
$S_1P_2$: Single-stranded template–primer complex [-]
*T*: Temperature [K]
$$T_{amb}$$: Ambient temperature [K]
$T_{ann}$: Temperature at annealing zone [K]
$T_{den}$: Temperature at denaturation zone [K]
$T_{ext}$: Temperature at extension zone [K]
$$t_R$$: Residence time [s]
$$\mathbf{u}$$: Velocity vector [m/s]
$$U_{in}$$: Inlet velocity [m/s]
$$V_{S}$$: Substrate volume [$$m^3$$]
*W*: Width [m]

## Code availability

Any code or file developed during the current study are available from the corresponding author on reasonable request.
